# Needs and expectations for artificial intelligence in emergency medicine according to Canadian physicians

**DOI:** 10.1186/s12913-023-09740-w

**Published:** 2023-07-25

**Authors:** Kyle W. Eastwood, Ronald May, Pantelis Andreou, Samina Abidi, Syed Sibte Raza Abidi, Osama M. Loubani

**Affiliations:** 1 Department of Emergency Medicine, Dalhousie University, 1796 Summer Street, Halifax Infirmary, 4Th Floor Emergency Department Administration Office, Halifax, NS B3H 2Y9 Canada; 2 Department of Community Health and Epidemiology, Dalhousie University, Halifax, Canada; 3 NICHE Research Group, Faculty of Computer Science, Dalhousie University, Halifax, Canada

**Keywords:** Artificial Intelligence, Needs-analysis, User-centered design, Emergency medicine, Survey

## Abstract

**Background:**

Artificial Intelligence (AI) is recognized by emergency physicians (EPs) as an important technology that will affect clinical practice. Several AI-tools have already been developed to aid care delivery in emergency medicine (EM). However, many EM tools appear to have been developed without a cross-disciplinary needs assessment, making it difficult to understand their broader importance to general-practice. Clinician surveys about AI tools have been conducted within other medical specialties to help guide future design. This study aims to understand the needs of Canadian EPs for the apt use of AI-based tools.

**Methods:**

A national cross-sectional, two-stage, mixed-method electronic survey of Canadian EPs was conducted from January-May 2022. The survey includes demographic and physician practice-pattern data, clinicians’ current use and perceptions of AI, and individual rankings of which EM work-activities most benefit from AI.

**Results:**

The primary outcome is a ranked list of high-priority AI-tools for EM that physicians want translated into general use within the next 10 years. When ranking specific AI examples, ‘automated charting/report generation’, ‘clinical prediction rules’ and ‘monitoring vitals with early-warning detection’ were the top items. When ranking by physician work-activities, ‘AI-tools for documentation’, ‘AI-tools for computer use’ and ‘AI-tools for triaging patients’ were the top items. For secondary outcomes, EPs indicated AI was ‘likely’ (43.1%) or ‘extremely likely’ (43.7%) to be able to complete the task of ‘documentation’ and indicated either ‘a-great-deal’ (32.8%) or ‘quite-a-bit’ (39.7%) of potential for AI in EM. Further, EPs were either ‘strongly’ (48.5%) or ‘somewhat’ (39.8%) interested in AI for EM.

**Conclusions:**

Physician input on the design of AI is essential to ensure the uptake of this technology. Translation of AI-tools to facilitate documentation is considered a high-priority, and respondents had high confidence that AI could facilitate this task. This study will guide future directions regarding the use of AI for EM and help direct efforts to address prevailing technology-translation barriers such as access to high-quality application-specific data and developing reporting guidelines for specific AI-applications. With a prioritized list of high-need AI applications, decision-makers can develop focused strategies to address these larger obstacles.

**Supplementary Information:**

The online version contains supplementary material available at 10.1186/s12913-023-09740-w.

## Background

Artificial Intelligence (AI) is the science and engineering of enabling computers to solve problems traditionally requiring human decision-making.^1^ Within Emergency Medicine (EM) physicians recognize that AI will have an immense impact on patient care [1, 2]. EM is one of a few specialties that manages both acute and sub-acute, undifferentiated patients, of all ages. With such heterogeneity, there are many different potential ways in which AI can augment care in the Emergency Department (ED). In ‘generalist’ specialties, such as EM, it is not always apparent what the most high-yield uses of AI are for the near future.

Over the last decade, an increasing number of original research studies and scoping reviews have been published that outline AI-tools for the ED. These articles describe multiple motivations for ED AI applications, for example, to improve patient safety through AI-enabled patient monitoring; to increase the speed and accuracy of triage, or the diagnosis and prognosis of a range of diseases or clinical-syndromes; to aid in targeted medication delivery; to augment imaging interpretation; and many others [3–6].

Despite the growth of clinical AI-tools, there are many obstacles to the implementation of AI-technology in medicine. These include concerns about the responsibility for medical-error related to AI, public perception, legal regulation, and the “black-box” phenomena or lack of ‘explainability’ of how the AI reach conclusions [1]. Moreover, from an adoption perspective, there is a lack of input from medical professionals in the needs assessment and later design of such applications [1, 6, 7]. To address this limitation, qualitative surveys have been conducted in specialties outside of EM to assess the needs of medical professionals that would benefit from new AI-tools [8–14]. Specifically, these studies explore physicians’ understanding and concerns about the technology, quantify their expectations, and identify needs that could guide the development of AI-tools.

A similar needs-analysis of AI use in EM does not exist. Several literature surveys summarize the current developments and applications of AI in EM, while commenting on its potential future benefits [1, 15–17]. Yet, few insights exist about how EPs currently use AI, their understanding of this technology, and importantly, how they want AI to be used in the clinical workflow or where they believe AI design efforts should be focused.

The primary aim of this study is to determine which EM work-activities are the highest priority for new AI-tool development in the near future. Secondary aims include identifying Canadian EPs’ understanding of AI, to gauge how AI is used in their practice, and to quantify their beliefs about the impact of AI on EM. Answering these questions will help address the need for additional user-input in the development of AI for ED applications.

## Methods

This study is a cross-sectional mixed-method electronic survey of Canadian physicians practicing EM, conducted in the spring of 2022. The original survey is included in Appendix A, and was implemented electronically using *Opinio* (ObjectPlanet Inc., Norway)*,* a secure online platform.

Participants were contacted using the Canadian Association of Emergency Physicians (CAEP) research-survey distribution service and the Society of Rural Physicians of Canada (SRPC) listserv. Residents, fellows and staff physicians practicing EM in Canada were surveyed. The study aimed to target 365 respondents (5% margin of error and 95% confidence interval) from an assumed total population of 3431 physicians calculated from the Royal College (RC) medical workforce database [18]. Assuming a 20% response rate to the survey, a minimum of 1820 participants were targeted to be invited to the survey. CAEP sent a total of three email blasts, spaced 1-month apart, to its 1494 subscribed members, and SRPC sent one email blast to its 350 members. The enrollment period was 4-months. The results are anonymous, and un-linked to the respondents’ identifying information. Participants could optionally enroll in a prize-draw including five gift-cards of fifteen Canadian dollars. The study was approved by the Research Ethics Board of Dalhousie University (File No. 1026940).

A survey draft was developed from similar studies in other medical specialties [8–12]. In addition, questions that aim to measure ‘technophilia’ - one’s enthusiasm for technology - were included from the TechPH scale, a validated instrument [19]. The development of additional original questions are described below.

First, a list of EM ‘work-activities’ performed by senior doctors was identified. This list was adapted from a systematic review by Abdulwahid et al*.* that proposes a classification system for EP work-activities [20]. Second, a list of existing AI-tools for EM was generated from scoping reviews; the final list was determined by consensus from the authors [21–25]. These two lists were used in two separate sections of the survey as outlined below. First, respondents were asked about their awareness and prior use of the AI-tools from these lists; second, respondents were asked to rank the priority of AI-tools on these lists.

Following iterative revisions, the survey was pilot tested on ten local EPs for written feedback (two FRCPC-EM [Fellow of the Royal College of Physicians of Canada, Emergency Medicine] residents, two Pediatric EM staff, two CCFP-EM [Certification in the College of Family Physicians, Emergency Medicine] staff physicians, and four FRCPC-EM staff physicians); the group had a balanced distribution of biological sex, and senior and junior staff.

The final survey is divided into four sections: (Section-I) Demographics; (Section-II) Secondary Outcomes: Knowledge and Comfort with Technology; (Section-III) Primary Outcome: Opportunities for AI-Tools in Patient Care in EM; and (Section-IV) Secondary Outcomes: Beliefs and Opinions about AI Impact and Significance.

The de-identified data was analyzed to extract summary statistics, and descriptive statistics to outline physicians’ rankings. The TechPH index, a composite score describing ‘technophilia,’ was calculated from the TechPH scale included in Section-II; see Appendix B for details.

Section-II includes both the EM ‘work-activities’ list and the list of existing AI-tools. Here, respondents were asked to indicate their awareness and prior use of these examples.

Section-III, measuring the primary outcome, asked participants to rank their top three choices from the same two lists of EM ‘work-activities’ and existing AI-tool items; an item’s total rank was calculated by weighted-sum, see Appendix B for details.

Analysis to assess rank-order preference, and co-variate analysis, were completed using the methodology outlined by Lee et al. [26]. The following variables were selected *a-priori* to assess if they significantly impact rank-order preference: Province of practice, hospital setting, prior educational focus (engineering or computer science versus other), TechPH index, prior clinical or research experience with AI, and years in practice.

Responses to open-ended questions were grouped and summarized in Appendix C. All statistical analysis was completed using R-Statistical-Software (R Foundation for Statistical Computing, Austria).

## Results

The enrollment period of four months was reached before the target of 365 responses. 1844 physicians were invited to participate, 230 physicians enrolled in the survey, and 171 completed all questions.

Table 1 summarizes the demographic, training, and clinical characteristics of respondents. Appendix D, Table 2, shows details on employment status and time-in-practice of respondents. Approximately half (53.6%) of respondents were within their first 10 years of practice.

**Table 1:** Physician Demographic Data

	**Total**	**Percent**
Training Pathway (*n* = 221)		
CCFP	20	9.0%
CCFP-EM	79	35.7%
CCFP with Enhanced EM Skills​	10	4.5%
FRCPC-EM	68	30.8%
Pediatrics-EM-	25	11.3%
Other	19	8.6%
Training Status (*n*=221)		
Training Complete	185	83.7%
Training in Progress	36	16.3%
Professional Position (*n* = 227)		
Resident	33	14.5%
Fellow	4	1.8%
Staff Physician	165	72.7%
Department Head	14	6.2%
Program Head	7	3.1%
Other	4	1.8%
Age (years, *n* = 218)		
< 30	20	9.2%
31–40	69	31.7%
41–50	57	26.1%
51–60	45	20.6%
61–70	22	10.1%
> 71	5	2.3%
Employment Location (*n* = 219)		
Alberta	25​	11.4%​
British Columbia	22​	10.0%​
Manitoba	14​	6.4%​
New Brunswick	12​	5.5%​
NewFoundland and Labrador	3​	1.4%​
Northwest Territories	2​	0.9%​
Nova Scotia	42​	19.2%​
Nunavut	1​	0.5%​
Ontario	64​	29.2%​
Prince Edward Island	7​	3.2%​
Quebec	22​	10.0%​
Saskatchewan	3​	1.4%​
Yukon	1​	0.5%​
I do not work in Canada	1​	0.5%​
Setting of Primary Clinical Practice (*n* = 219)		
Rural / Remote ED	5​	2.3%​
Regional ED	20​	9.1%​
Community ED	42​	19.2%​
Tertiary Care ED	67​	30.6%​
Teaching ED	56​	25.6%​
Pediatric ED	29​	13.2%​

**Fig. 1:**
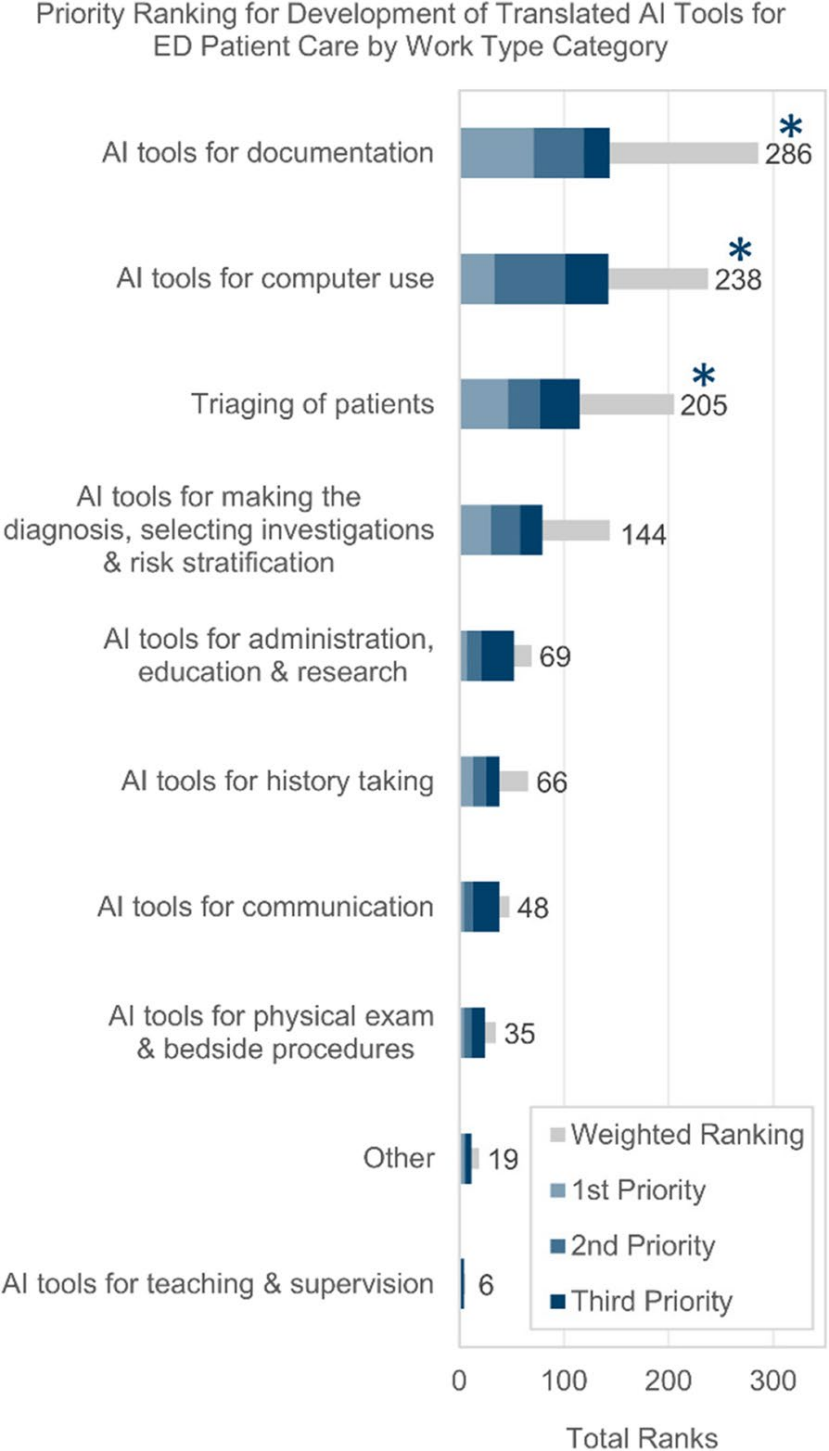
Canadian Emergency Physicians’ rankings of AI-tool examples by work-type; Survey Question - which of the following are highest priory for developing a fully translated AI-tool for patient care in the next 10 years? (*n* = 186)

The primary outcomes of this study are the top priorities for new ED AI-tool development in the next 10 years. Figure 1 demonstrates the priority ranking for new AI-tools categorized by work-activities based on physician opinion. The top three items are ‘AI-tools for documentation’, ‘AI-tools for computer-use’ and ‘AI-tools for triaging of patients.’ These ranks were statistically significant, and there was no variation in rank-order with the sub-group analysis. The ‘AI-tools for computer-use’ work-type focuses on ease-of-use of computer systems employed in the ED; open-ended comments from respondents include “optimizing [and] simplifying EMR workflows to limit human cognitive demand”; and “reduce [the] time used in an EMR, fewer clicks”; and “current test entering [is] awkward and time consuming”.

**Fig. 2:**
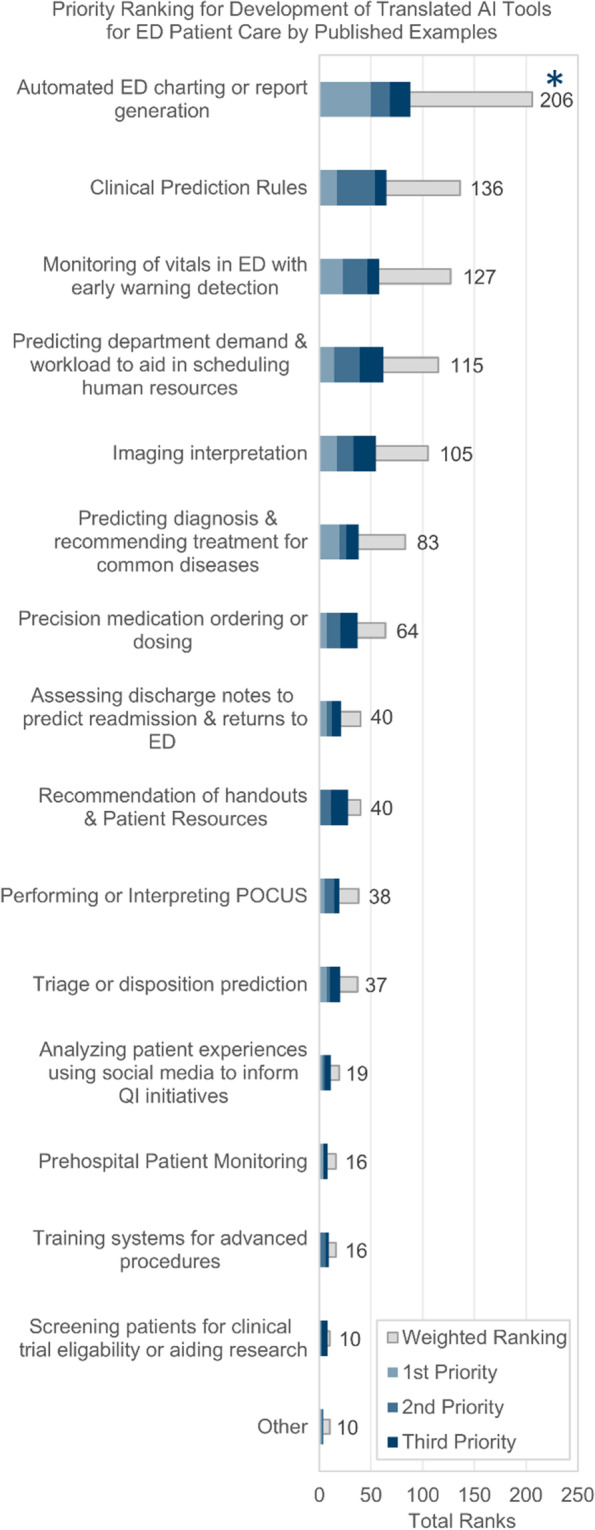
Canadian Emergency Physicians’ rankings of existing published AI-tool examples; Survey Question - which of the following are highest priory for developing a fully translated AI-tool for patient care in the next 10 years? (*n* = 177)

Figure 2 depicts the top priorities when respondents were asked to rank example AI-tools instead of general work-activities. ‘Automated charting or report generation’ was the first-priority and was statistically significant. Other highly ranked items include ‘AI-powered clinical prediction rules’ (Clinical Decision Rules, CDR), ‘monitoring of vitals with early warning detection’, ‘predicting department demand and workload’, ‘imaging interpretation’ and ‘predicting diagnoses’. The differences between these rankings were not statistically significant, and there were no changes in rank-order in the sub-group analysis. See Appendix D, Table 3 for details.

Participants comfort with technology was low-moderate, with 33.0% identifying as technology-enthusiasts, and 20.3% as technology-hobbyists. 7.5% and 5.7% of respondents previously studied either computer-technology or information-technology, respectively. 4.4% have previously studied engineering and 2.2% computer-science. See Appendix D, Table 4 for details.

The participants mean rating of ‘Technophilia’ based on the TechPH index was a moderate 3.30 (std 0.65) on a range of one (high technology-anxiety) to five (high technology-enthusiasm).

Examples of participants definitions of AI are summarized in Appendix C. In the authors’ opinion, 23.6% of respondents answered correctly and 45.8% had partially correct definitions.

Respondent’s experience with AI was low-moderate; the results in Appendix D Table 5 indicate 38.2% have ‘read journal articles about AI in general’, and 45.2% have ‘read journal articles about AI in medicine’. Most respondents indicated “very-little” to “some” experience with AI in their personal lives, clinical work in general, work in EM and in research.

Appendix D Table 6 includes the same items from Figs. 1 and 2., however this question asked respondents to comment on their past awareness and usage of these items. The most common ED physician work-activities in which participants ***have used*** AI include ‘AI-tools for computer use’ (29.1%), ‘AI-tools for documentation’ (20.1%) and ‘AI-tools for administration/education/research’ (16.9%). The most common work-activities where EPs ***have heard-of*** AI-tools, but not necessarily used the tools, include ‘AI-tools for making the diagnosis/selecting investigations/risk-stratification’ (42.9%), ‘triaging of patients’ (42.9%), and ‘AI-tools for computer use’ (40.2%).

Framing this question in another way, the most common examples of published AI-tools that EPs ***have used*** in practice include ‘AI-powered clinical prediction rules’ (51.8%), ‘AI-powered monitoring of patient vitals and early warning-systems’ (29.9%), ‘AI-powered PoCUS’ (25.4%), and ‘AI-powered recommendations of patient handouts/resources’ (20.8%). Interestingly, the most common examples of published AI-tools that EPs ***have heard-of***, not necessarily used themselves, are ‘AI-powered XRAY’ (64.0%), ‘CT’ (60.9%), ‘MRI’ (55.8%) and ‘US interpretation’ (54.3%).

Regarding EPs’ opinions about AI’s impact on physicians’ jobs over the next 10 years, 60.6% of respondents believe, because of the impact of AI, “jobs will change slightly” while 35.9% believe “jobs will change substantially”.

Further, the responding EP indicated a high potential for AI in EM (32.8% ‘a great deal of potential’, 39.7% ‘quite a bit of potential’, 24.7% ‘some potential’), and indicated high personal interest in AI for ED patient care (48.5% ‘strongly agree’, 39.8% ‘somewhat agree’).

In terms of how the job may change, respondents felt AI is most likely able to complete the following tasks: ‘Provide documentation’ (43.7% Extremely likely, 43.1% likely), and ‘formulate personalized medication/therapy plans’ (13.8% extremely likely, 50.0% likely). Respondents were neutral regarding AI’s ability to ‘analyze patient information to reach a diagnosis’, ‘reach a prognosis’, ‘formulate personalized treatment plans’ or ‘evaluate when to refer to a specialist’. Physicians indicated it was ‘extremely unlikely’ (45.4%) or ‘unlikely’ (36.2%) for AI to be able to provide empathetic care. See Appendix D, Table 7 for details.

## Discussion

This study outlines EPs work-activities that are the highest priority for new AI-tool development. Survey participants were asked to consider the development of a fully translated AI-tool for patient care that would be available at most EDs in Canada in the next 10-years. To triangulate responses, participants were asked to rank a list of common ED work-activities that may benefit from AI, and a list of existing AI-tool examples.

The survey sampled 5.65% of Canadian physicians practicing emergency medicine, not including residents, based on 2019 data from the RC [18]. This estimate does not account for physicians practising EM with other licence types; for example, CCFP physicians without additional EM designations. Additionally, 6.07% of trainees were surveyed (33 of an estimated 543 active residents); based on the 2019 residency quotas [18]. In general, the breakdown of survey respondents fit with national trends. For example, responses by licence-type are similar to the RC reported proportions; however, this survey had slightly higher PEM representation. The age distribution of survey respondents is slightly older than the RC reported proportions, with 57.4% less than 44 years old in the general population and 40.9% of survey respondents less than 41 years old.

Considering geography, there was a disproportionately high response from the Maritimes (29.2%); the author’s practice location being Halifax. However, the remaining geographic distribution is consistent with the RC database; the other highest response rates come from Ontario (29.2%), Alberta (11.4%) and Quebec (10.0%), which contain approximately 38%, 17.5% and 16.3% of the target population, respectively. The high response result may also relate to each region having large AI institutes (Vector, Amii, Mila, respectively) with provincial strategies for AI adoption. The results are also biased towards urban practitioners, with only 11.4% practicing in rural or regional centers; important input from rural physicians may been missed.

Concerning familiarity with technology in-general, respondents were neutral; approximately half neither “dislike” nor “like” technology and 9.0% indicated “no interest in technology.” The average TechPH index agrees with the finding that most respondents were neutral regarding technology interest [19]. A measure of the baseline ‘technophilia’ of Canadian EPs for comparison is unknown. Overall, these outcomes are reassuring that the respondents include general EPs and are not necessarily biased towards physicians hyperspecialized in technology development, nor are they actively opposed to the integration of new technology. Compared to the average Canadian, our study population may be more cautious regarding the use of AI for healthcare. A 2018 survey from the Canadian Medical Association (CMA) of 2000 adult Canadians found that 69% believe AI could be the solution to the challenges facing our healthcare system; 70% thought that using more technology for personal healthcare would prevent disease, and 50% indicated they would seek-out doctors who use AI in their practice [27].

Respondents have low overall experience with AI in their personal lives, clinical roles or work as EPs. We speculate that the ‘low’ personal experience with AI may relate to misconceptions about the technology, as we assume that most Canadians are daily consumers of AI-enabled apps and productivity tools (weather, navigation, search-engines, voice-to-text). This response may also be from the framing and interpretation of the question.

When asked about their understanding of AI-technology, 87.2% of respondents “agree” or “strongly-agree” they “understand what is meant by AI.” However, only 23.6% of respondents had a completely correct definition of AI (see Appendix C). These results suggest that more education around the concept and purpose of AI may be needed.

Few respondents have conducted any research in AI (4.5%). This result agrees with follow-up questions, where most respondents indicated “no experience at all” (71.4%) or “very little experience” (11.1%) with AI-research. Again, these findings corroborate the neutral TechPH index. However, almost all respondents either “somewhat agree” (39.8%) or “strongly agree” (48.5%) that they are interested in AI for EM.

Overall, EPs agree that AI has potential use for EM; however, physicians feel there will be only “slight” (60.6%) to “moderate” (35.9%) impact on how their job will change. This result suggests physicians believe AI will enhance current roles but not disrupt the specialty over the next 10 years. This opinion is consistent with findings from surveys of psychiatrists and family-physicians [9].

Considering EPs’ impressions about AI’s capabilities, most thought AI was “likely” or “extremely likely” to be able to provide documentation. Interestingly, much of the current focus of EM AI development aims at tasks such as reaching a prognosis, formulating a treatment plan, formulating personalized medications and evaluating when to refer to a specialist, despite these being ranked either “neutral” or “likely.” Providing empathetic care was ranked as “extremely unlikely” or “unlikely”. These findings also match the opinions of psychiatrists and family-physicians surveyed with the same instrument [9, 12].

Respondents also indicated examples where they “have used” or “heard of” AI being used in EM (Table 6). They were provided specific examples of AI-tools, and for triangulation, also general ‘work-activities’ where AI is used. Of note, the ranks of “have used” for ED work-activities, do not map to the AI-tool example ranks; the first choice AI-example ‘clinical prediction rules’ maps to the fourth choice AI-work-types ‘AI-tools for making the diagnosis, selecting investigations, etc.’. One explanation is that physicians may not agree on how to classify different types of AI-tools, or there are other more important AI-tools within the work-type categories not listed in the examples.

As well, interpreting Table 6 in the context of Table 7, many of the “have used” items fit into the “prognosis/diagnosis” and “formulating a treatment plan” categories, which are all areas that physicians have guarded opinions about. Interestingly, the ED-documentation-tools have only been used by 13.2% of respondents and only 41.1% had heard of AI-tool examples. Yet, this was the task with the best perception of being accomplished by AI. Additionally, all categories of AI-imaging interpretation were ranked low in terms of past use by EPs; a surprising result given the large body of AI-research for radiology. Perhaps the use of this technology by Radiologists is not immediately obvious to the EP receiving the reports.

For the study’s primary outcome, there is clear consensus for translation of AI-tools to facilitate documentation, and as mentioned, respondents had the most confidence that AI could facilitate this task. Although many new ED information systems (EDIS) have some AI integration, as indicated, few respondents “have used” or “heard of” ‘automated charting or report generation’ and only 37% have “heard of” and 20.1% “have used” AI-tools for documentation. Based on the responses, we would suggest that tools for documentation are prioritized to meet both the expectation and needs in EM.

The emphasis on ‘AI-documentation’ is not unique to EM, with recent surveys of primary-care providers, psychiatrists as well as a heterogenous population of US-clinicians also strongly indicating that AI could aid clinical documentation [9, 12, 13]. There is clear evidence that clinical documentation is both time-consuming and a source of burn-out for all physicians [28, 29]. However, the current summaries of AI-applications for EM do not clearly emphasize ED-documentation as a large category for active AI development [1, 15–17]. Although clinical-documentation may be considered general to all specialties, the environment of the ED will generate unique user-requirements, and therefore ED-documentation should be included as an EM specific application for AI development initiatives.

The ‘documentation’ category is broad, including electronic charting with voice-to-text, or active listening with AI-powered scribes, or AI-powered summaries of patient records to consolidate them into succinct and accessible formats. Future work should clarify these needs in detail, perhaps using focused interviews.

Separate from these specific recommendations, this survey provides insights into a potential strategy for implementing AI tools in an ED setting. As such, we recommend that (i) ED physicians be engaged in the specification, design, evaluation, and implementation of future AI driven tools; (ii) Priority should be placed on developing proof of concept AI-solutions for the high-yield problems identified by ED physicians; (iii) Solutions should embed AI tools within the ED’s existing digital infrastructure and clinical workflow; and (iv) Developers should identify measurable and impactful outcomes for AI use, and use standardized metrics to assess these outcomes.

In conclusion, AI in an ED setting can be seen as an innovation agent, as the analysis of ED data can generate new insights about the effectiveness of certain procedures/policies and lead to the optimizations of ED resources. AI is not here to change ED practices, rather it offers solutions to optimize a number of practice challenges. The survey responses clearly point to perceived value for AI in the ED, however certain activities are more amendable to AI driven support. For instance, automated charting particularly using speech recognition and transcription, rapid interpretation of real-time ED data for clinical decision support, patient risk stratification, and forecasting for staffing. The opportunity to benefit from AI based applications relies heavily on their integration within the current clinical workflows and the data sources used by ED physicians. This will ensure that ED physicians do not need to change their practice to make use of AI tools, rather AI driven support is seamlessly available at the point of care. Overall, the growth of AI in medicine is on the rise and it is fair to conclude that the use of AI in ED is quite near in the future.

### Limitations

Study limitations are as follows: First, this survey reflects the Canadian perspective and may not be generalizable internationally. Further, there is a sampling bias towards physicians subscribed to CAEP and a selection bias towards physicians interested in AI, and those practicing in the Maritimes. Further, the sample size is less than the apriori target of 365 respondents for representing the Canadian Emergency Physician population; the four-month deadline and maximum allowable three-survey blasts were reached before complete enrollment. The study is also limited by the confounding effects of variables not measured. Additionally, the survey’s questionnaire was not previously validated, despite being carefully designed. There is no standardized classification system for AI tools for emergency medicine, as such, some of the AI examples or physician work-types may be interpreted differently by respondents. For example, “clinical prediction rules” are synonymous with clinical decision rules (CDR) which are tools used to identify patients at higher risk for disease-specific clinical conditions, or are used to prevent the overuse of specific diagnostic testing [30]. While this is commonly understood in the Canadian Emergency context, the phrasing could be misinterpreted to mean prediction in general. As well, the study does not consider other health professionals working in EM.

### Future directions

There are many limitations in applying survey research methodologies. In addition to the known limitations of electronic surveys, specific to this study, there was confusion about the meaning of AI in-general and no opportunity for participants to clarify certain applications and items in the questionnaire. In the future, alternate methodologies including focused interviews and focus groups should be employed to further explore the themes identified in this study.

## Conclusions

User-centered design is essential to technology translation. A lack of physician input into AI development is a major translation barrier for these practice-changing AI tools. A survey of Canadian EPs has identified ‘automated charting or report generation’, ‘clinical prediction rules’ and ‘monitoring of vitals with early warning detection’ as high-priority areas for new development. This prioritization can aid policymakers in decision-making for AI data sharing, developing reporting guidelines and facilitating external validations studies for high-demand AI-tools.

## Supplementary Information

**Additional file 1.** Appendix A: Survey.

**Additional file 2.** Appendix B: Calculations.

**Additional file 3.** Appendix C: Open-Ended Responses.

**Additional file 4.** Appendix D: Supplemental Results.

## Data Availability

The datasets used and analyzed during the current study are available from the corresponding author on reasonable request.

## Abbreviations

AI: Artificial Intelligence
CAEP: Canadian Association of Emergency Physicians
CCFP-EM: Certification in the College of Family Physicians, Emergency Medicine
EDIS: Emergency Department Information System
EM: Emergency Medicine
EP: Emergency Physician (EP) / Emergency Physicians (EPs)
FRCPC-EM: Fellow of the Royal College of Physicians of Canada, Emergency Medicine
RC: Royal College
SRPC: Society of Rural Physicians of Canada
