# Supplementary figures and images for: Needs and expectations for artificial intelligence in emergency medicine according to Canadian physicians
Source: BMC Health Serv Res. 2023 Jul 25;23:798. doi: 10.1186/s12913-023-09740-w (PMC10369807; doi:10.1186/s12913-023-09740-w)

Appendix A: Survey

| 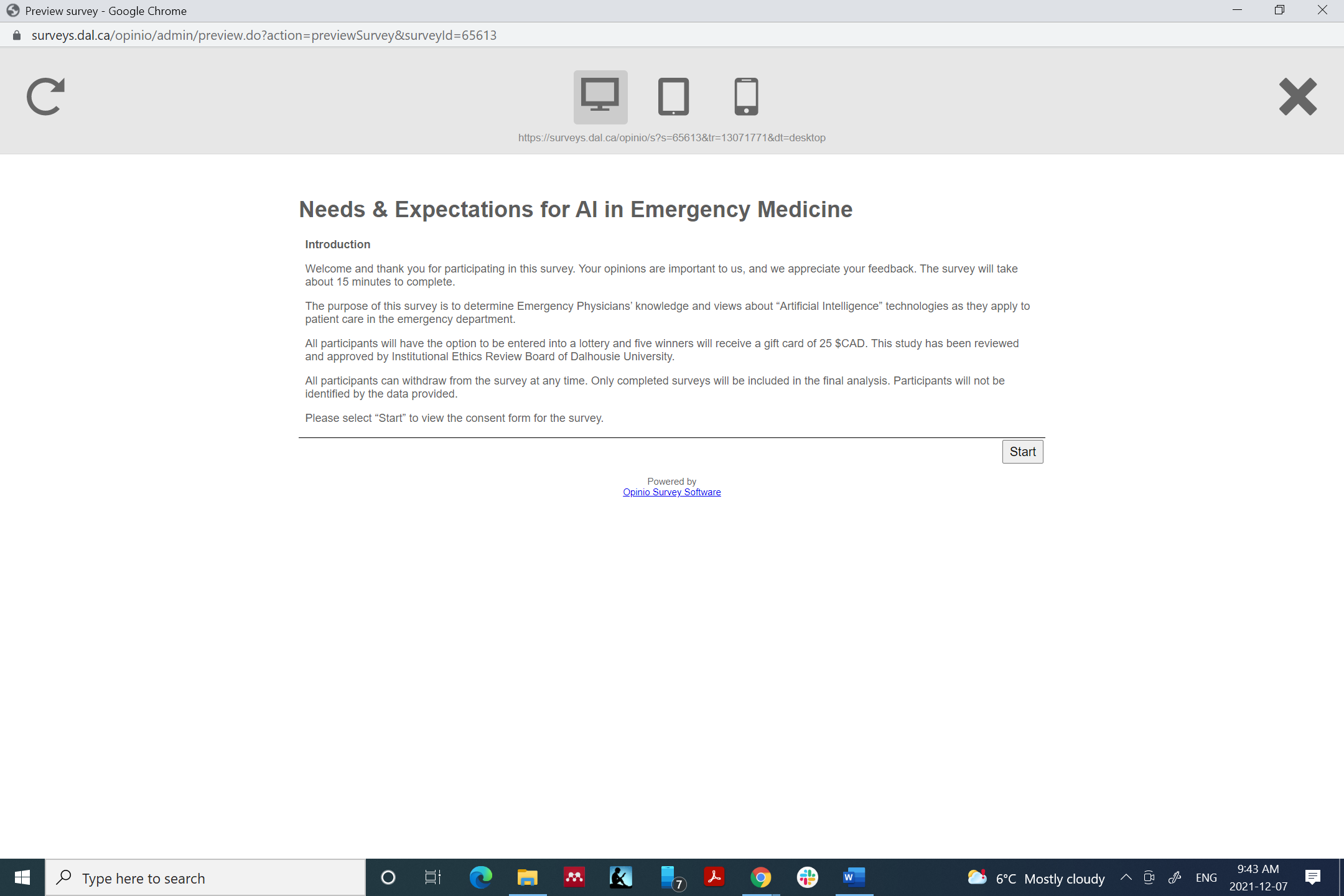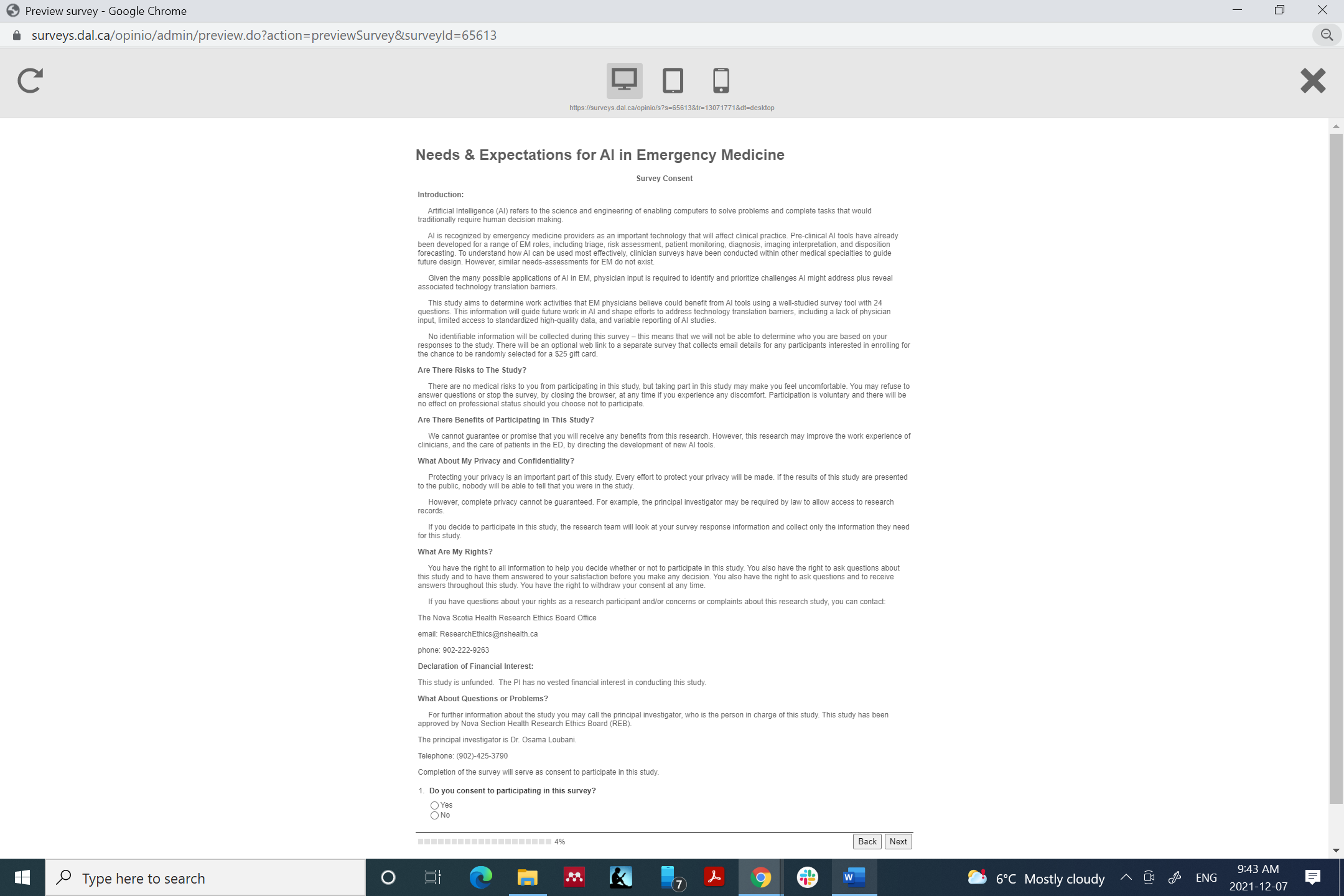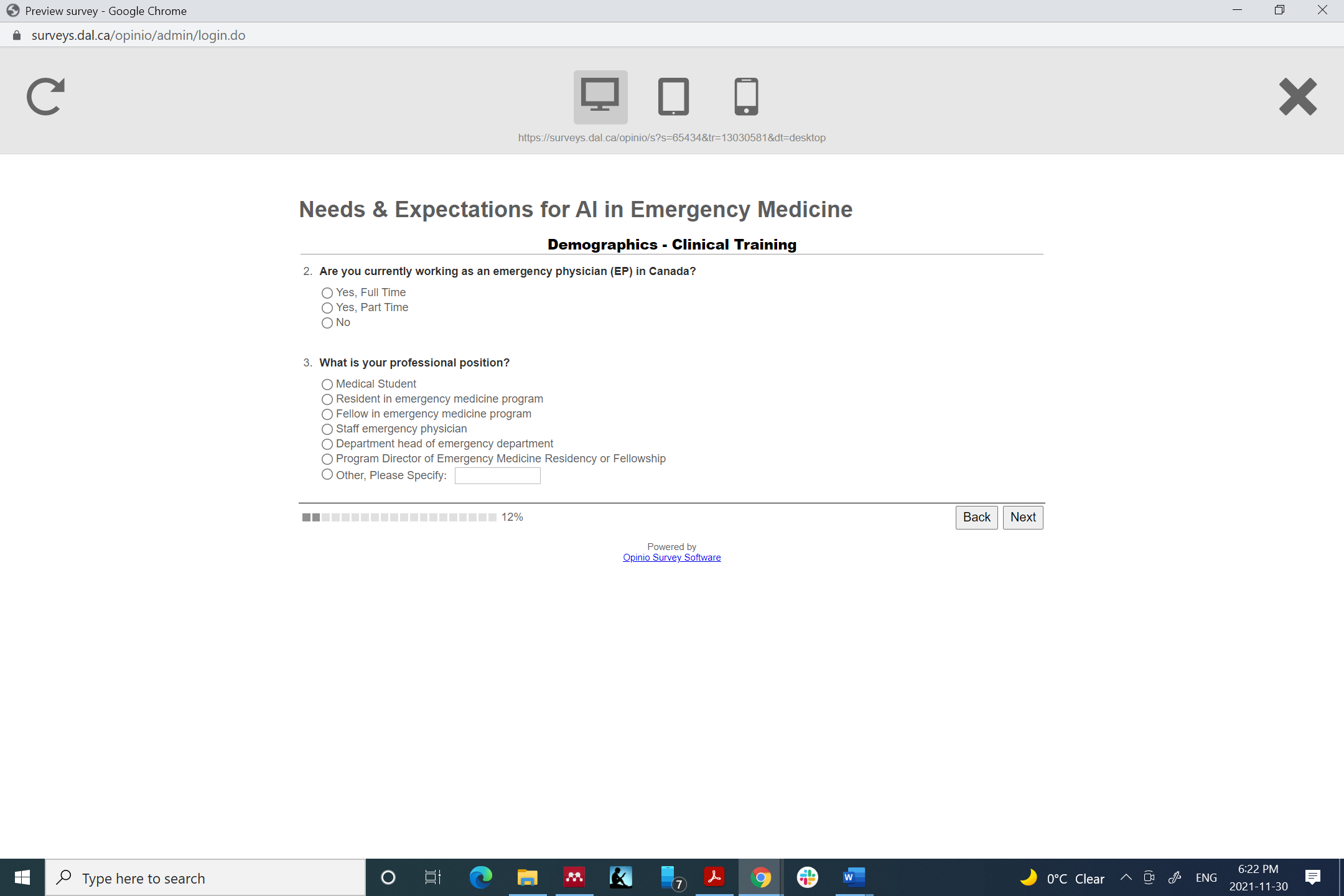 | 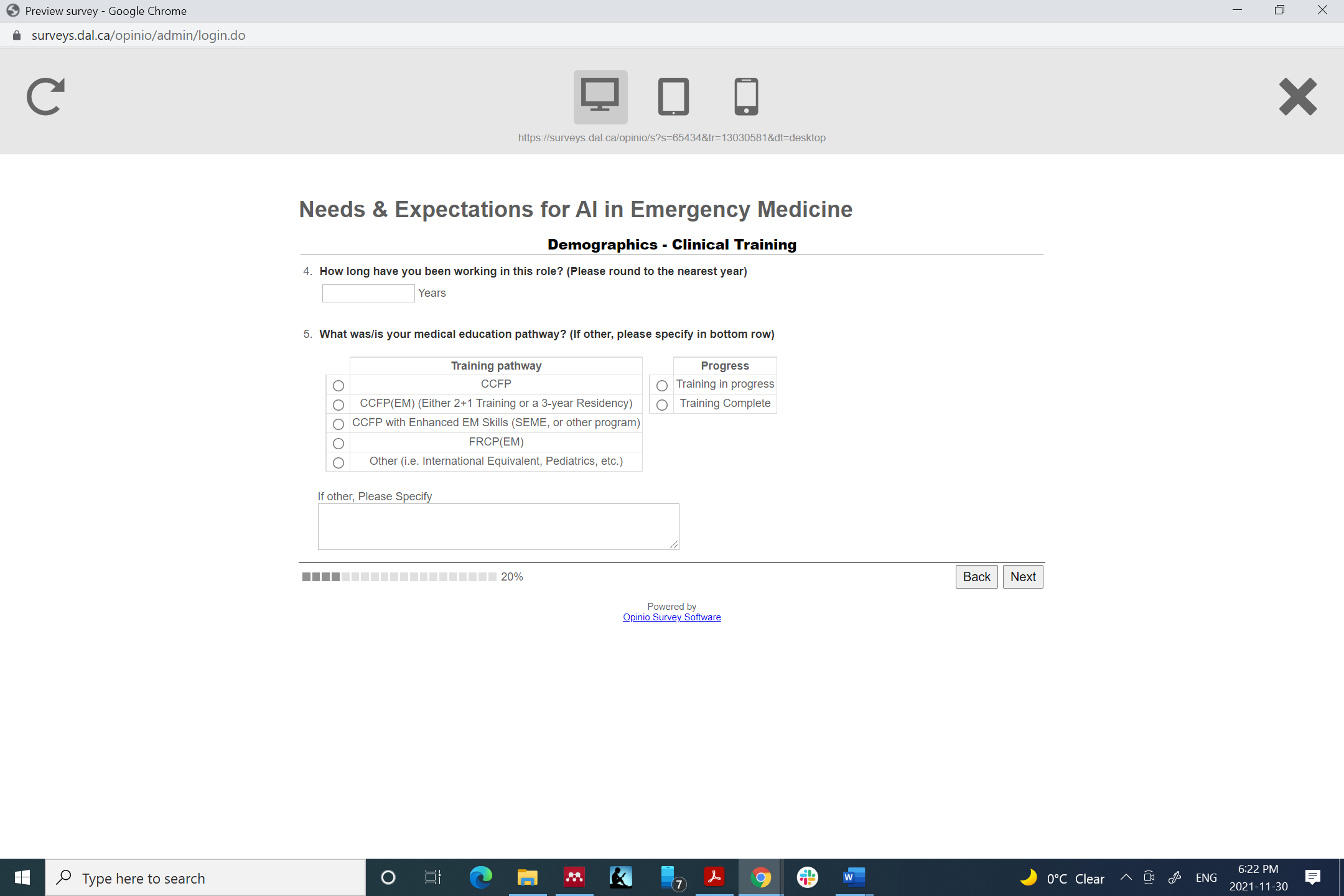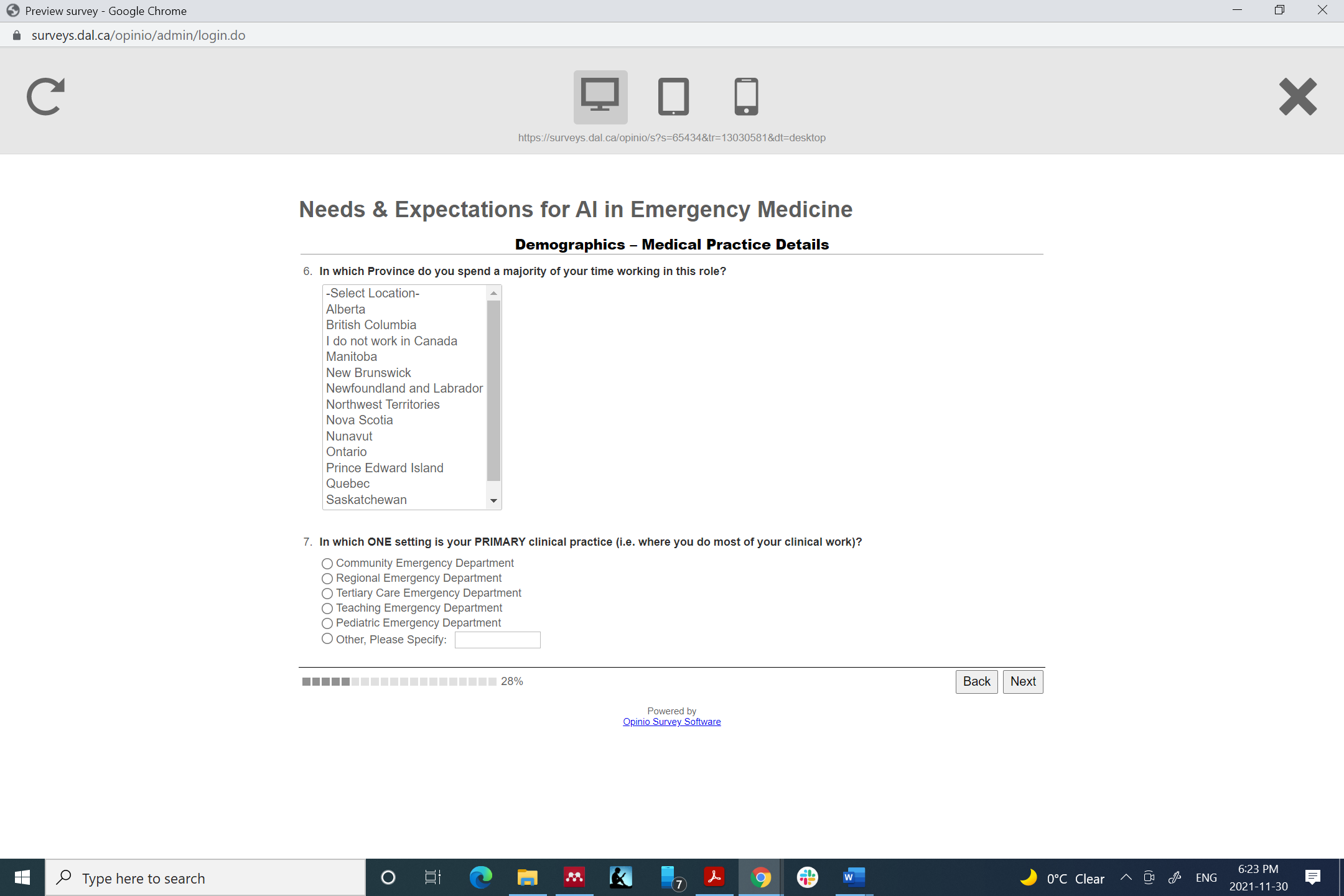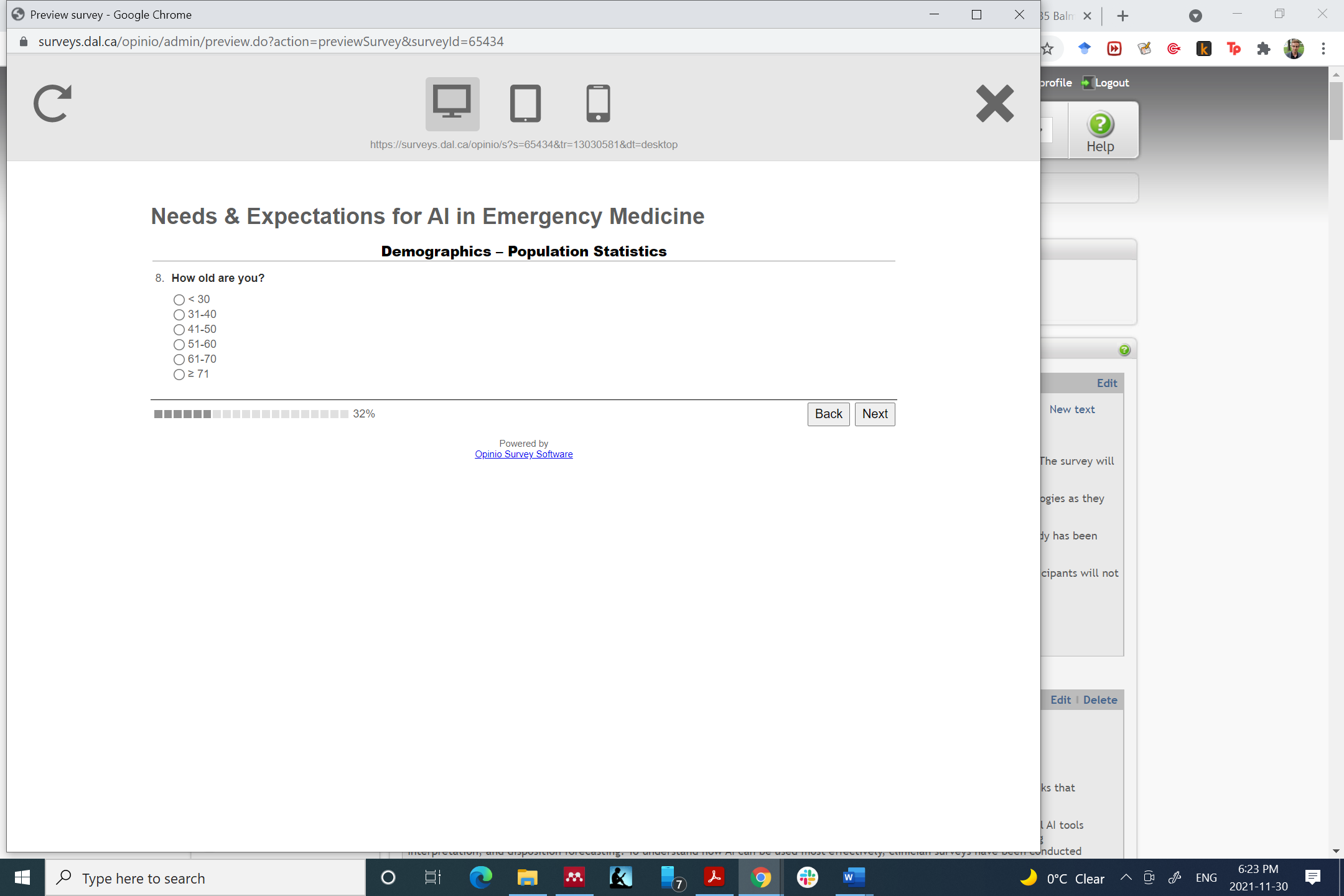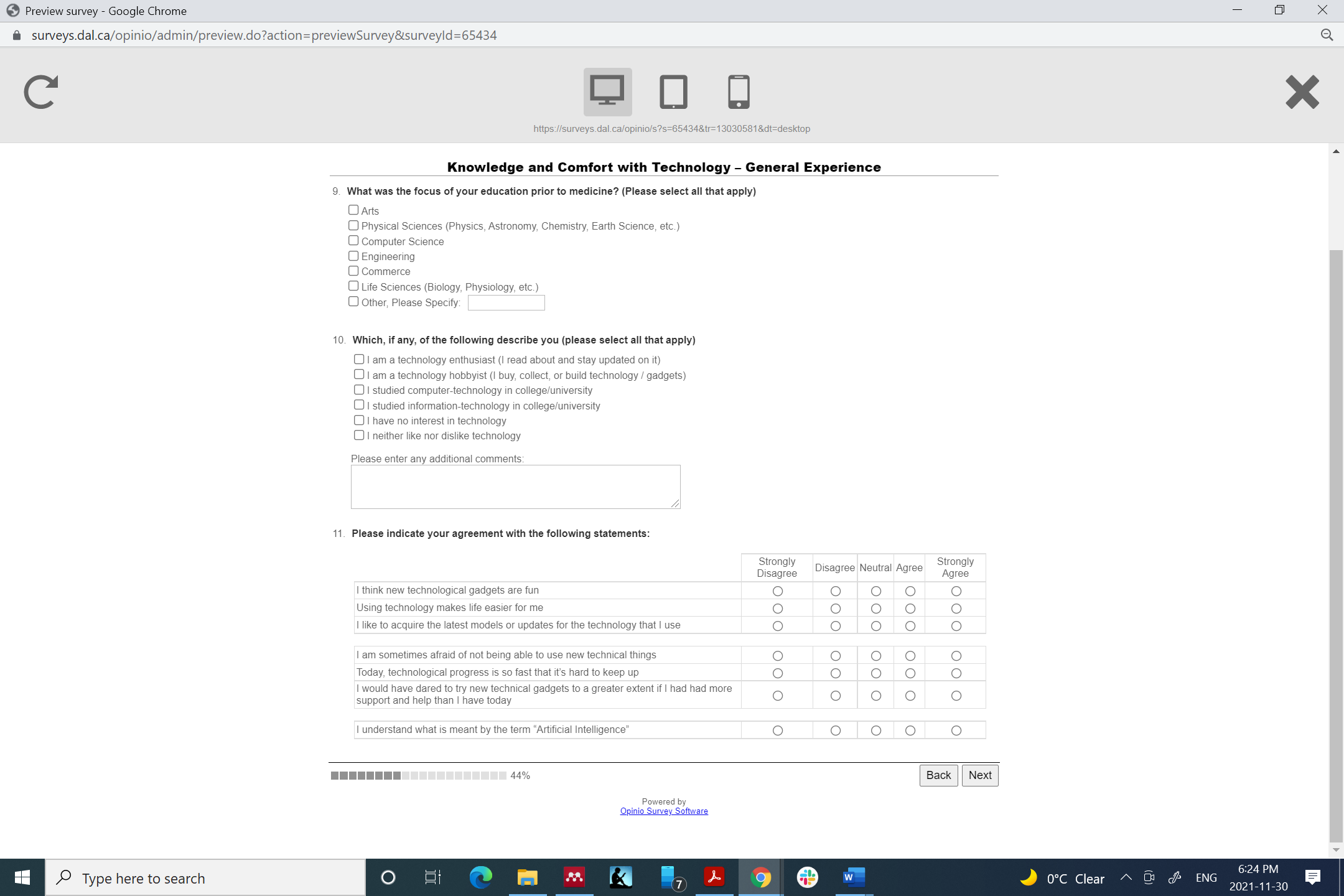 |
| --- | --- |
| 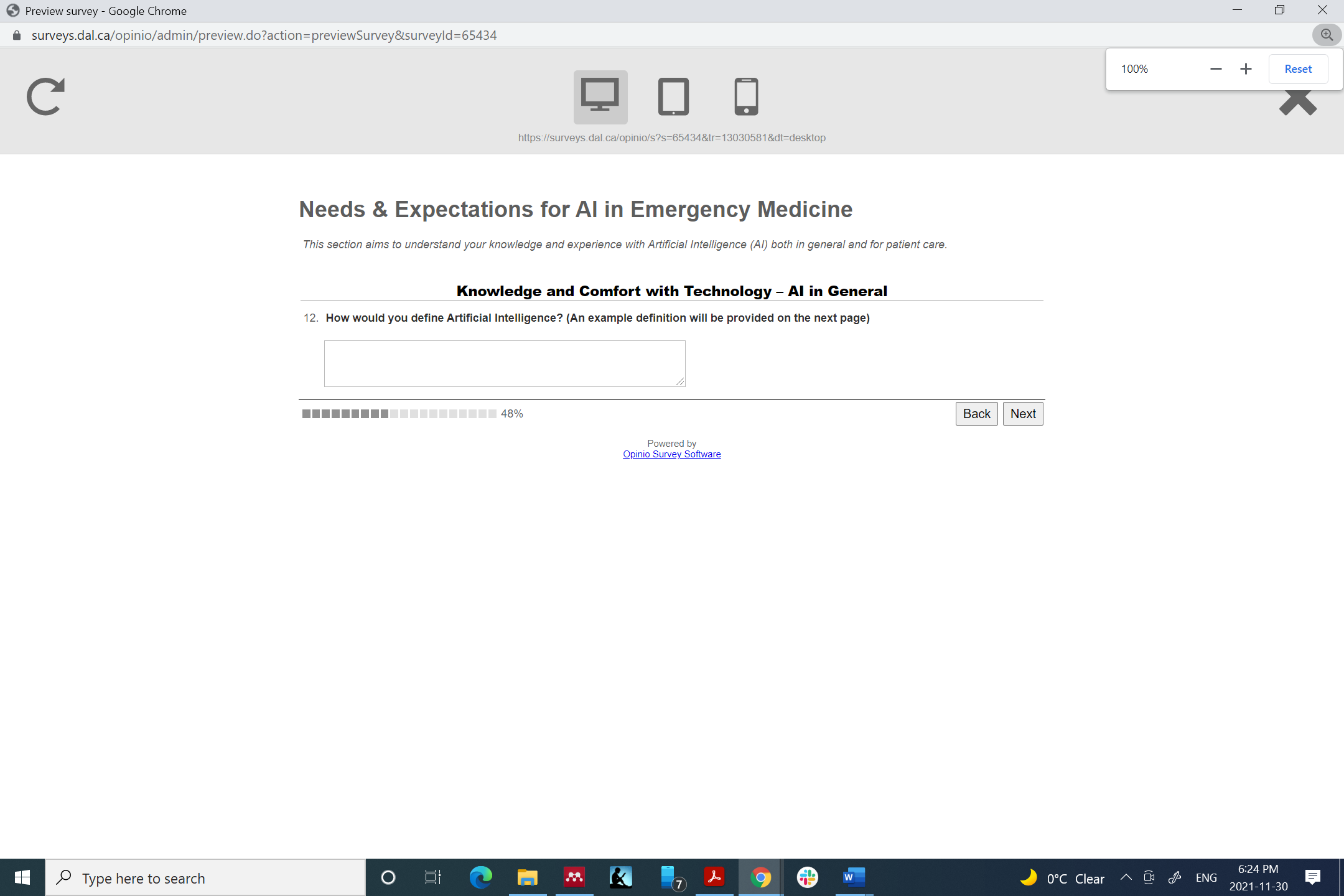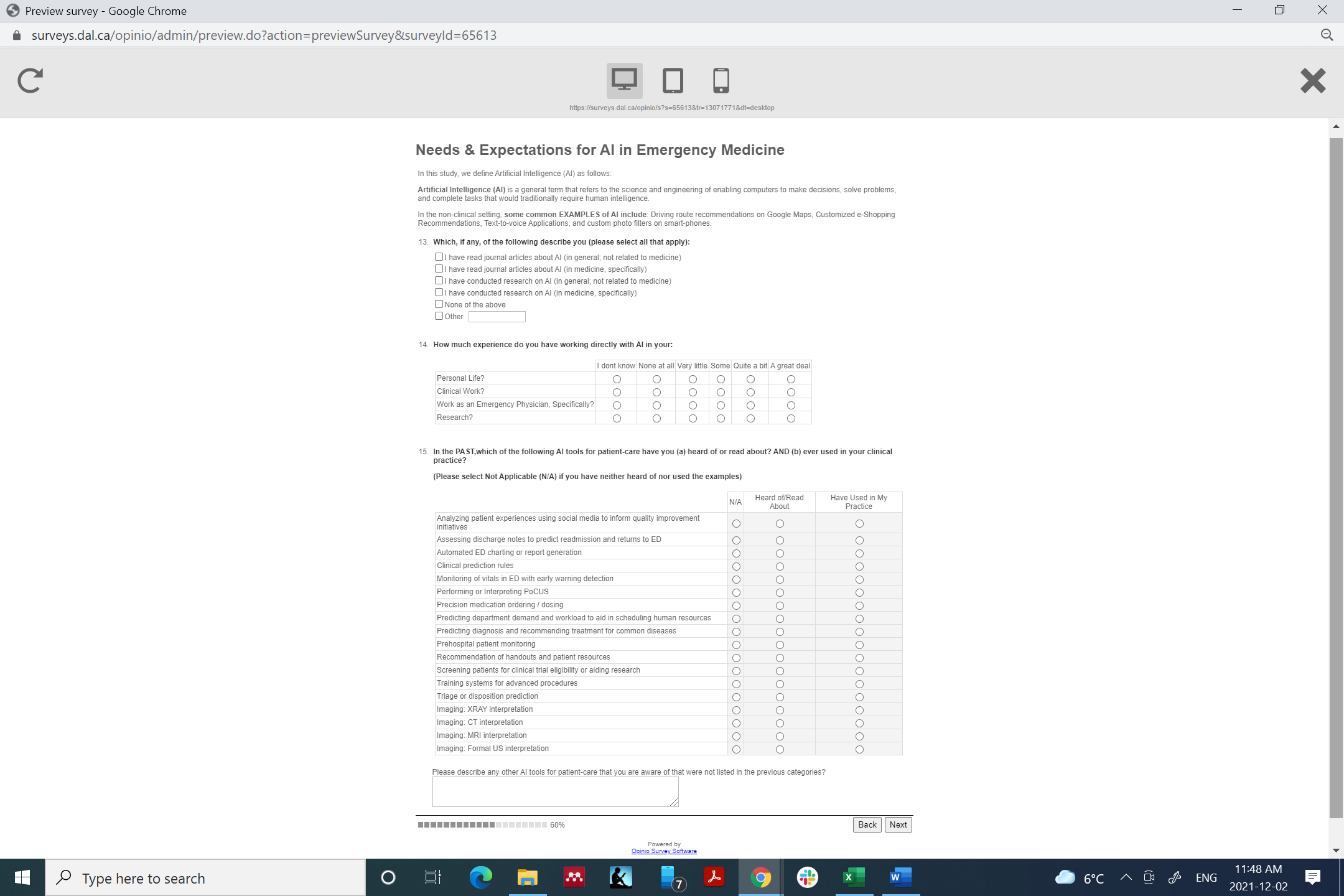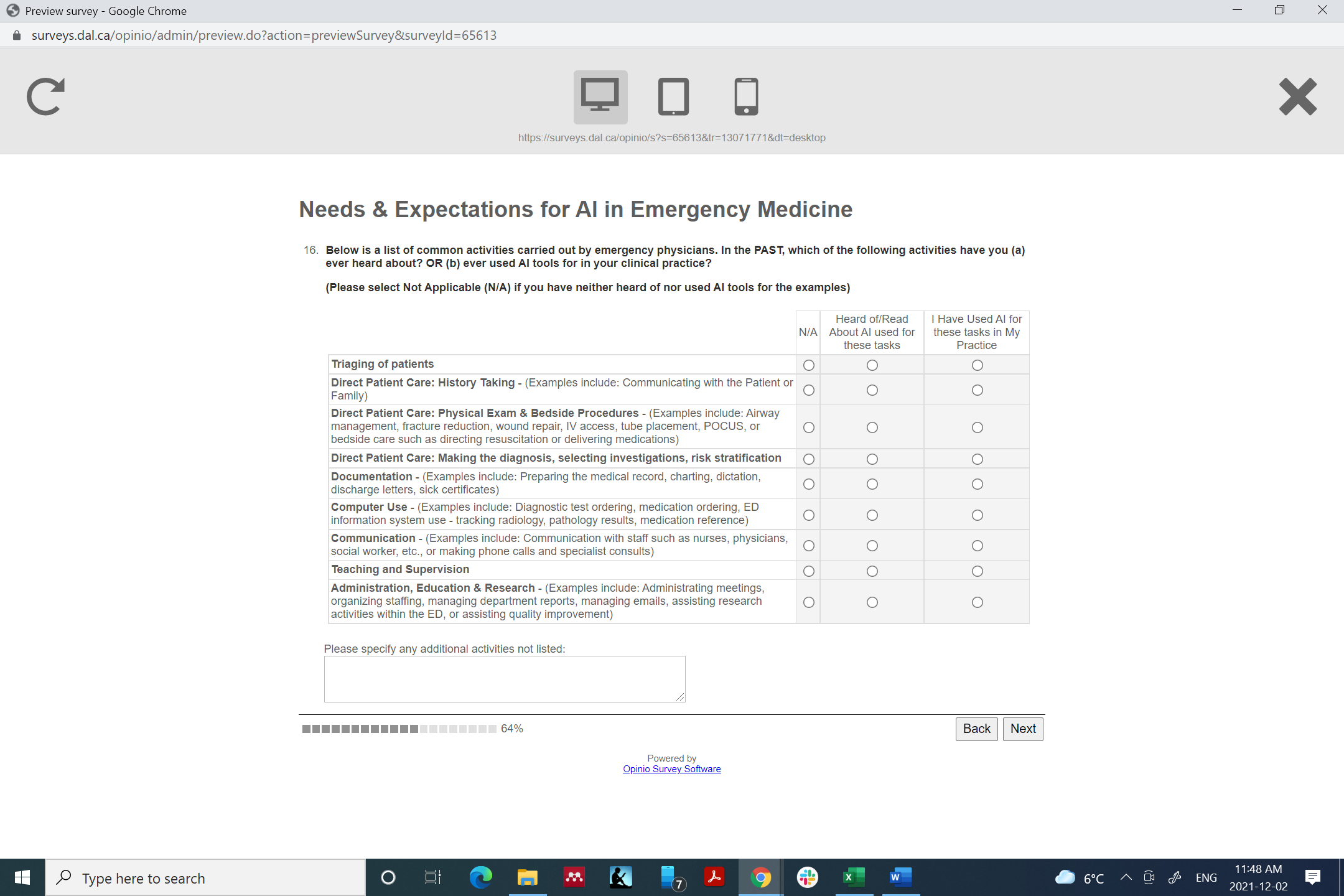 | 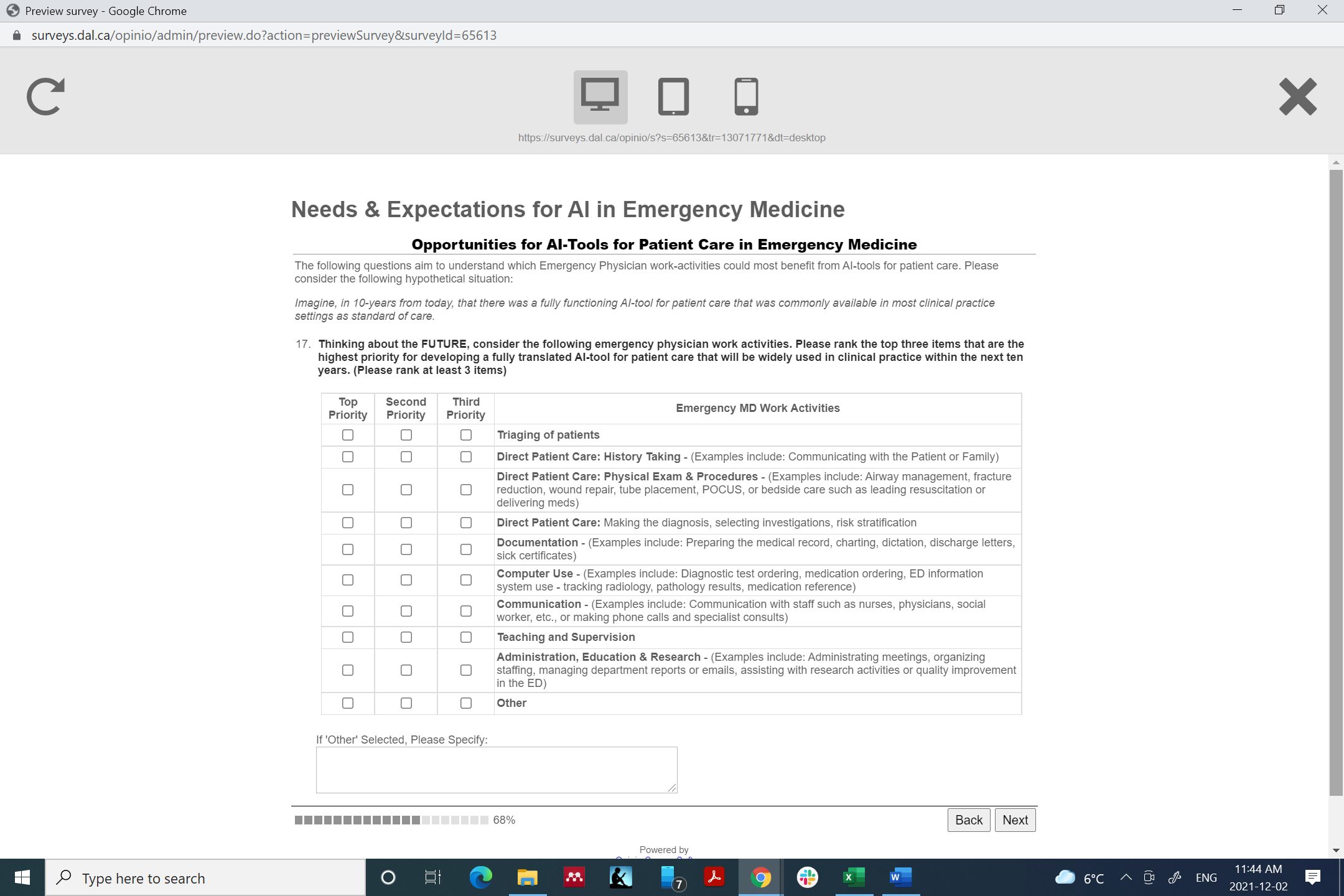  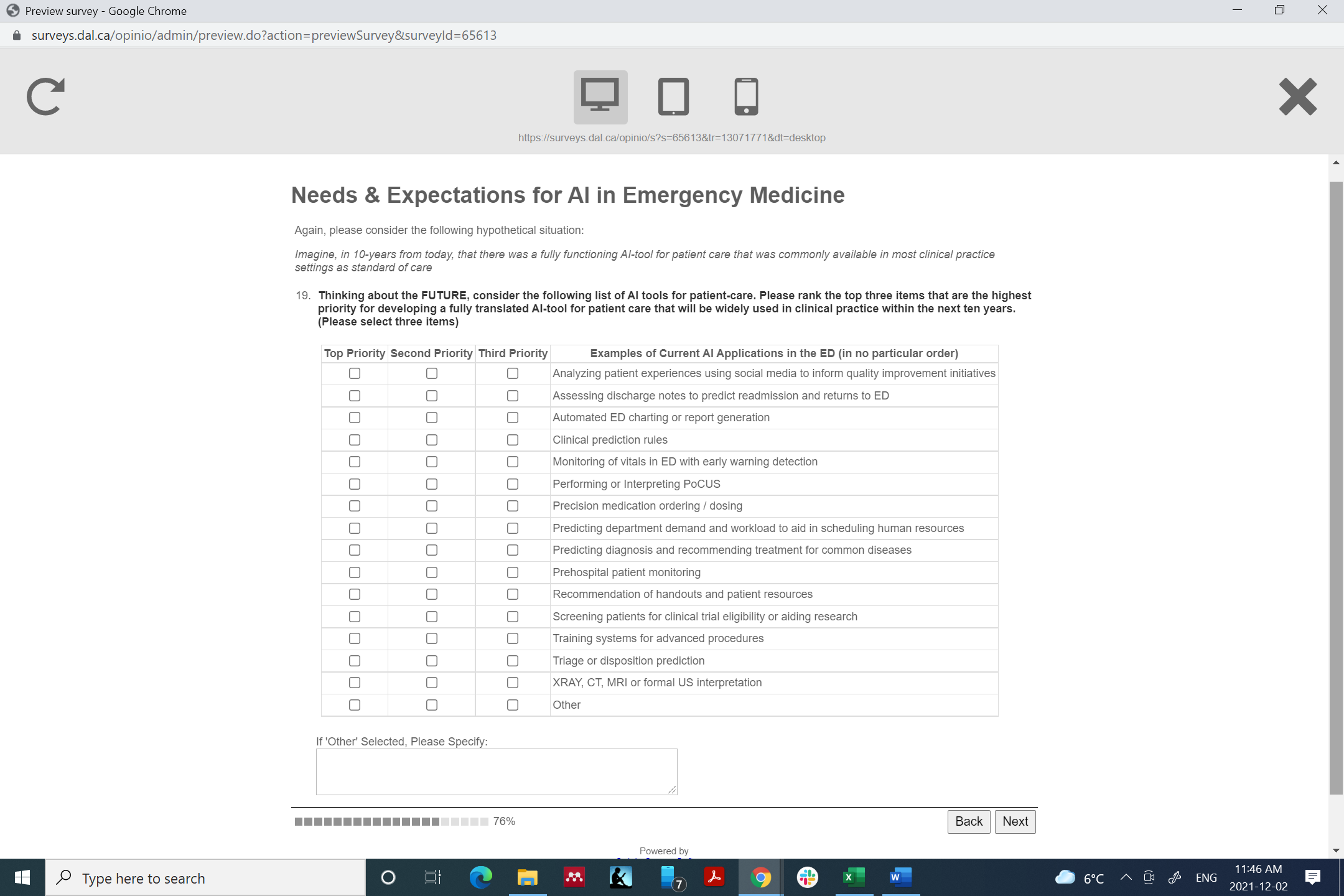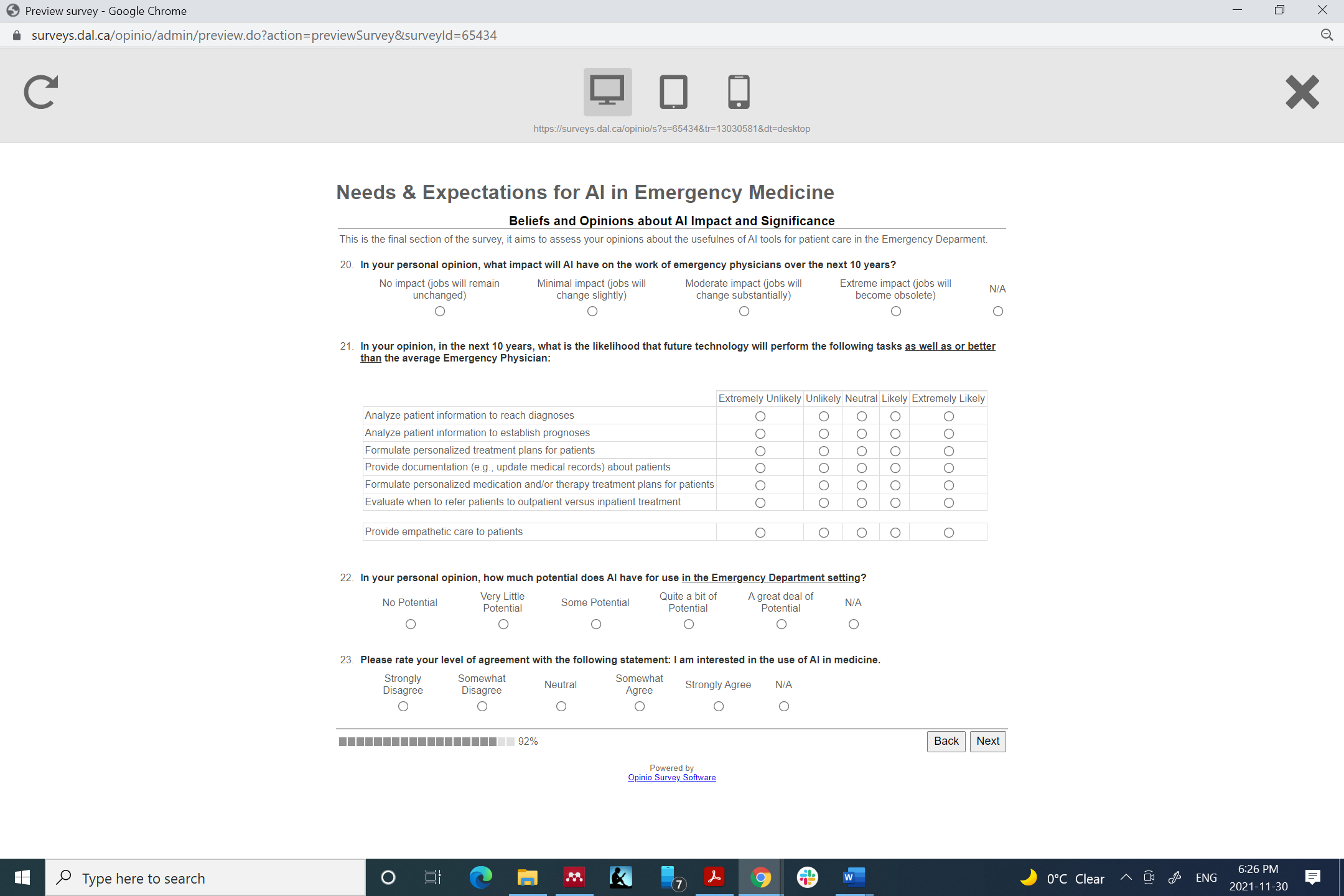 |
| 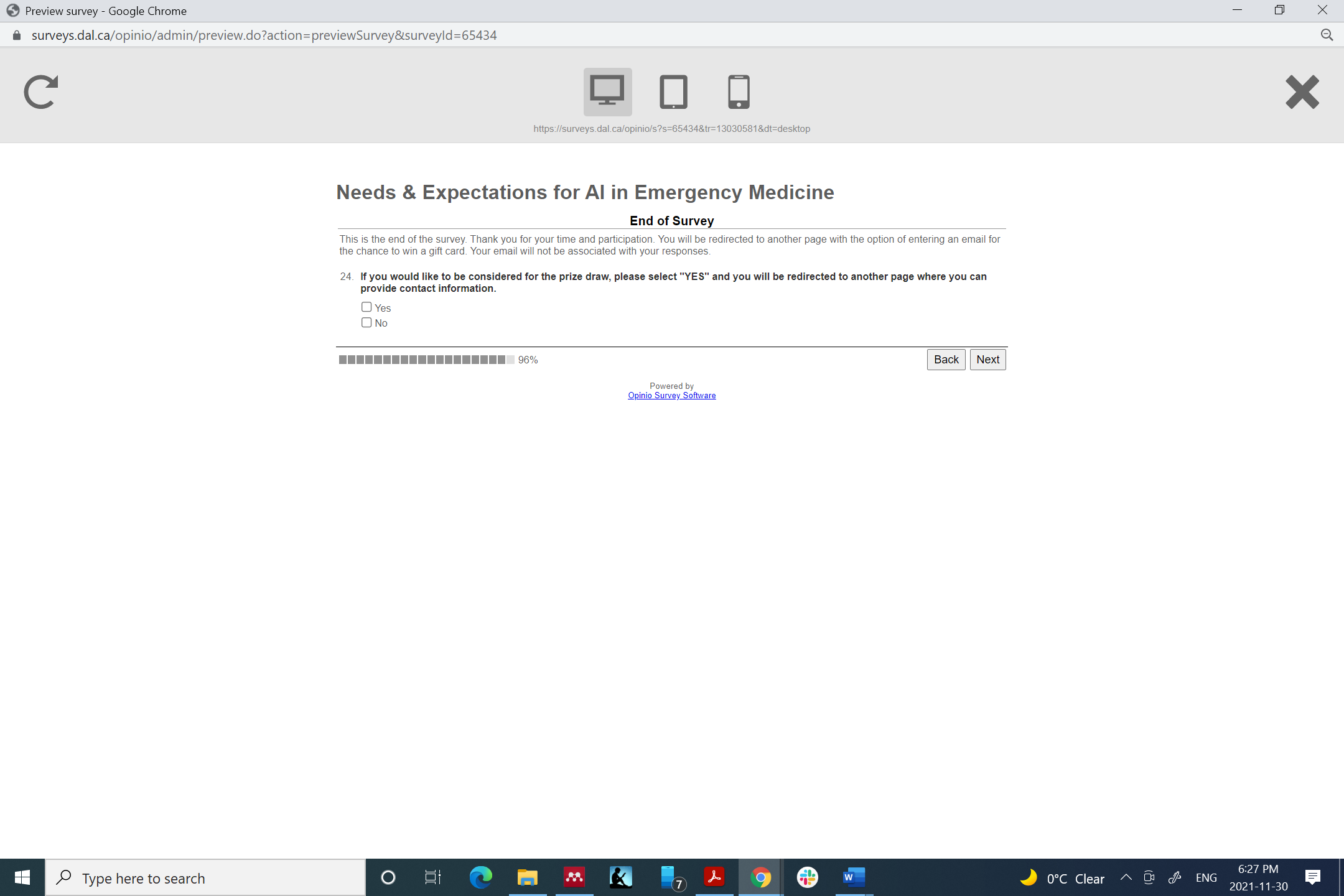 | 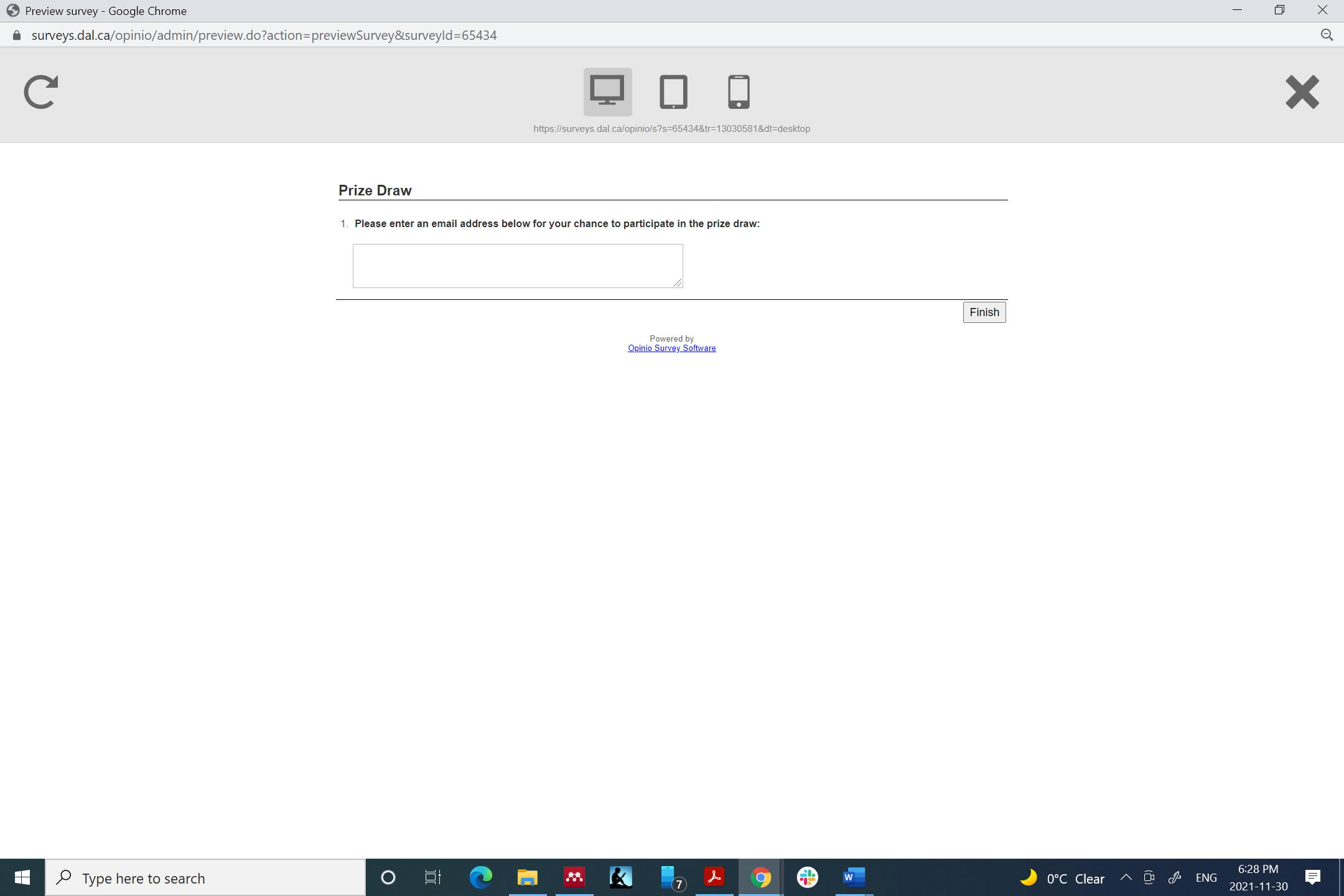 |

Supplement: Supplementary file 1 — Additional file 1. Appendix A: Survey. [file 12913_2023_9740_MOESM1_ESM.docx]
