# Supplementary material for: Needs and expectations for artificial intelligence in emergency medicine according to Canadian physicians
Source: BMC Health Serv Res. 2023 Jul 25;23:798. doi: 10.1186/s12913-023-09740-w (PMC10369807; doi:10.1186/s12913-023-09740-w)
Supplement: Supplementary file 2 — Additional file 2. Appendix B: Calculations. [file 12913_2023_9740_MOESM2_ESM.docx]

**Appendix B – Calculations**

**TechPH Composite Score:**

The TechPH is a composite score calculated from the following items ranked between ‘strongly disagree’ (1) and ‘strongly agree’ (5):

1. I think new technological gadgets are fun
2. Using technology makes life easier for me
3. I like to acquire the latest models and updates
4. I am sometimes afraid of not being able to use new technological things
5. Today technological progress is so fast its hard to keep up
6. I would have dared to try new technological things if I had more help

The composite score is calculated as:

$$TechPH=\frac{{Item}_{i}+{Item}_{ii}+{Item}_{iii}-{(Item}_{iV}+{Item}_{V}+{Item}_{vi})+18}{6}$$

**AI Item Priority Weighted Ranking Score:**

For each $j$ example of AI-Tool or physician work-activity, the weighted priority ranking is calculated by summing the frequency (denoted with subscript $f$) of the first priority, second priority and third priorities for that $jth$ example, each weighted by three, two or one, respectively. We assume there are $n$ respondents that completed the survey and the ranks from the $i^{th}$ respondent are binary values of either zero or one.

$${Item}_{j} Weighted Rank=\sum_{i=1}^{n} 3\left( {First Ranks}_{f} \right)+2\left( {Second Ranks}_{f} \right)+\left( {Third Ranks}_{f} \right)$$
