# Supplementary material for: Needs and expectations for artificial intelligence in emergency medicine according to Canadian physicians
Source: BMC Health Serv Res. 2023 Jul 25;23:798. doi: 10.1186/s12913-023-09740-w (PMC10369807; doi:10.1186/s12913-023-09740-w)
Supplement: Supplementary file 3 — Additional file 3. Appendix C: Open-Ended Responses. [file 12913_2023_9740_MOESM3_ESM.docx]

**Appendix C – Open-Ended Responses**

The following are the open-ended responses of emergency physicians when asked to define artificial intelligence. Of all 230 respondents who initiated the survey, 212 attempted this question, and 207 wrote a response. The responses were assessed by the authors by consensus: 50 (23.6%) were correct and included terms that reflected computers completing tasks previously requiring human intelligence. 65 (30.7%) of responses were incorrect and either were blank or provided a definition or task that applied to traditional computer programming. 97 (45.8%) of the responses had some but not all of the elements of the accepted definition.

The author’s accepted definition as shown in the survey:


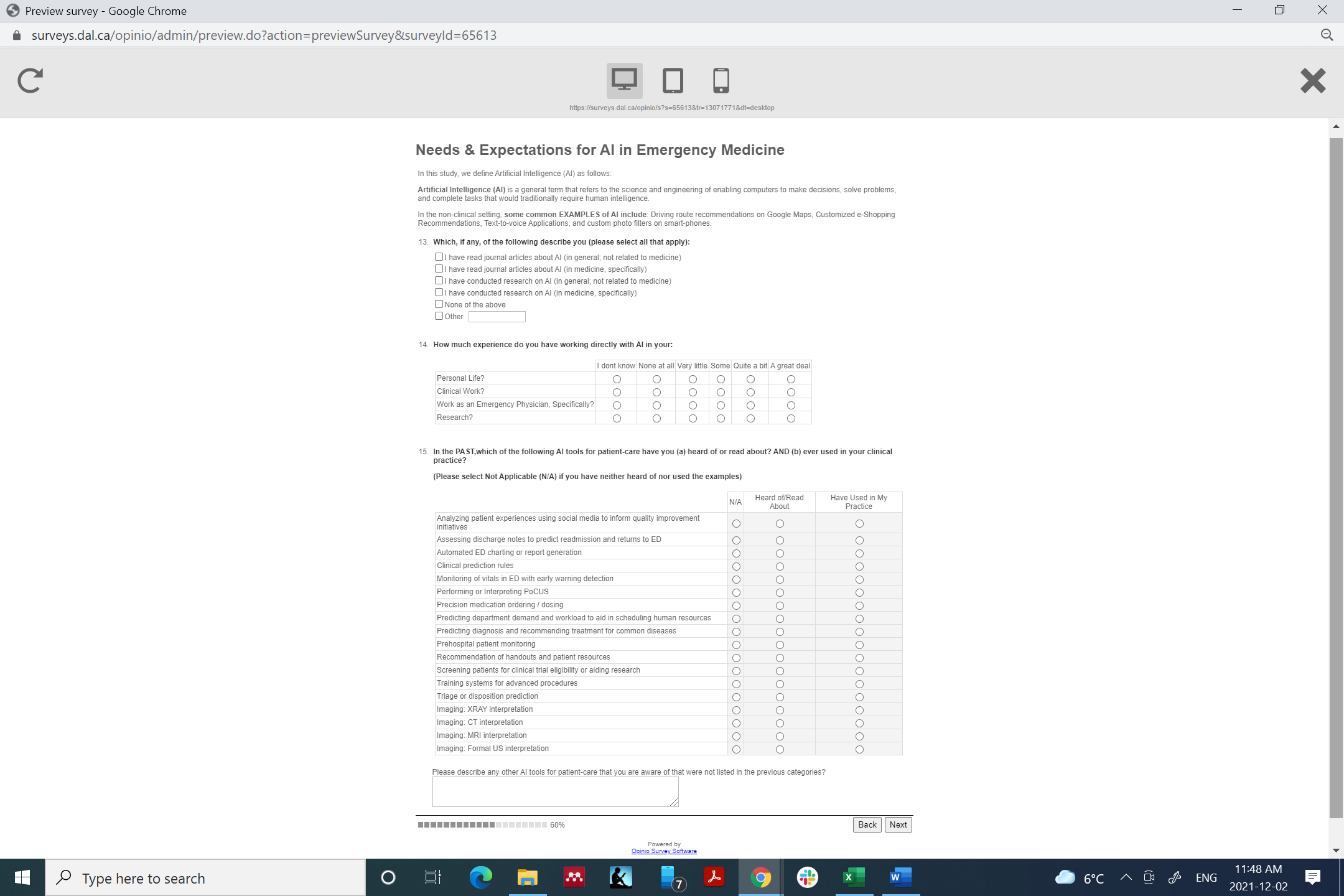


| **Respondent’s Answers to Question “Please define AI”** | **Correct** | **Too**  **General** | **Incorrect / Unsure** |
| --- | --- | --- | --- |
| **TOTAL SCORES:** | **(50)** | **(97)** | **(65)** |
| Computers completing complex tasks that previously could only be done by humans. | **✓** |  |  |
| Development of algorithms or systems able to perform tasks usually requiring human input/intelligence. | **✓** |  |  |
| Machines that support animal intelligence to produce work or to solve problems. |  | **✓** |  |
| The term is broad and can encompass anything from computer-aided support (e.g. decision-making support) to fully automated systems. Examples can include clinical AI to help at the bedside, scheduling-type AI (to learn and adjust manpower based on predictors of high volume), or AI built into EMR systems. In general, 'AI' implies some sort of learning algorithm - with alterations in the algorithm based on some sort of feedback mechanism or 'training'. | **✓** |  |  |
| The use of computational power to replicate human intelligence. | **✓** |  |  |
| The use of engineering design and applications toward using computers/computing power to solve problems or perform tasks that would otherwise be done by humans. | **✓** |  |  |
| Computer algorithms based on what is known about human behavior used to suggest/predict human decision making or problem solving. | **✓** |  |  |
| Artificial systems that can perform tasks that normally require human intelligence. | **✓** |  |  |
| Computer doing calculations or comparisons at rapid speed to develop answers. |  | **✓** |  |
| Simulation of human intelligence to perform tasks. |  | **✓** |  |
| Computer or machine-based learning through user generated algorithms and user generated data. |  | **✓** |  |
| Computer systems that perform tasks that usually require human intelligence and are able to learn. | **✓** |  |  |
| Technology that allows a machine to simulate human behaviour and intelligence - in medicine often refers to machine learning (using algorithms for learning rather than programming functions). | **✓** |  |  |
| Machines have the ability to adapt to new challenges and learn from them |  | **✓** |  |
| Not sure |  |  | **✓** |
| Algorithms that analyze data to perform a function and can learn and adapt. |  | **✓** |  |
| Computer analysis of data using algorithms to assess state of being, predict outcomes and recommend course of action. |  | **✓** |  |
| Deep learning by background running software that can interpret or perform tasks. |  | **✓** |  |
| Computer based decision making. |  | **✓** |  |
| Artificial intelligence describes any computer system that uses data to generate new information or predictive information. |  | **✓** |  |
| A system that has the ability to adapt and change outputs without following strict prescriptive algorithms. |  | **✓** |  |
| Robotics, machine learning, computer-based algorithms to support patient care. |  | **✓** |  |
| Decision making capabilities by machines based on programming and computer learning. |  | **✓** |  |
| The machine interpretation of multiple data points to facilitate decision making. |  | **✓** |  |
| Computers that are able to teach themselves and operate with a human prescribed set of rules for the greater good of humanity and the planet as a whole. |  | **✓** |  |
| Automation through technology that predicts outcomes based on certain algorithms. |  | **✓** |  |
| Computer-aided decision tool. |  |  | **✓** |
| The use of computer-generated algorithms and analytical techniques to answer questions using digital data. |  | **✓** |  |
| A program that allows for machine learning (trial and error that allows the computer to achieve a certain goal). |  | **✓** |  |
| The ability of computers to complete tasks and/or make decisions without significant human input. | **✓** |  |  |
| A system of taking in new information, attempting to find a pattern, and then adapting new attempts for the margin of error of previous attempts in hopes of creating a working model or appropriate end goal. |  | **✓** |  |
| Computer systems that are able to adapt based on ongoing evaluation of data. |  | **✓** |  |
| Systems of learning programmed into technology which become independent in terms of generating information/data. | **✓** |  |  |
| Technology that independently makes and enacts decisions based on input. | **✓** |  |  |
| Intelligence created by humans which has the capability to learn and adapt. | **✓** |  |  |
| Computer assisted decision making. |  |  | **✓** |
| Computer systems can collect large volumes of data, and with algorithms (i.e. machine learning) can develop predictions / create tools. |  | **✓** |  |
| Intelligence from a machine. |  | **✓** |  |
| Technology system that can use input to infer answers and solutions. Often uses machine learning to develop these answers. |  | **✓** |  |
| A computerized algorithmic process that learns as it goes along. |  | **✓** |  |
| Use of machine technology that is capable of refining its algorithms based on previous outcomes. |  | **✓** |  |
| This is a tough question... apologies but no answer. |  |  | **✓** |
| Computer algorithms (several kinds) often using big data to find solutions otherwise difficult to find together with the ability to 'learn' from multiple imputations of available data. |  | **✓** |  |
| Intelligence produced by machines (i.e. 'artificial' compared to natural intelligence of humans). |  | **✓** |  |
| A process whereby computers collate and analyze large amounts of data to identify correlations and trends then extrapolate from these to new (not yet correlated) data. |  | **✓** |  |
| A computer program(s) which is capable of 'learning' - improving it's performance on a task by a feedback loop that allows it to improve with experience. |  | **✓** |  |
| Computer systems that complete human tasks. |  | **✓** |  |
| A system built to take in information, process it through a set of algorithms / models trained w/ known data, and then generate needed information for a specific goal / purpose, resembling natural intelligence in humans. | **✓** |  |  |
| My understanding of artificial intelligence is that it refers to reasoning and decision making by technology rather than purely algorithmic functioning. | **✓** |  |  |
| Machine intelligence (as opposed to human intelligence). |  | **✓** |  |
| Use of complex computer methods- neural networks to 'learn' from repetitive activities. |  | **✓** |  |
| Machine learning. |  | **✓** |  |
| Use of algorithms to predict outcomes. |  |  | **✓** |
| A machine (computer) learns to do human tasks while learning and inputting new information to make those tasks more accurately and efficiently completed than a human could do to the computers ability to rapidly assimilate and keep information. | **✓** |  |  |
| Assistive technology meant to continually improve ability/interpretation of a certain task over time. |  | **✓** |  |
| Computers trying to think independently. |  | **✓** |  |
| Use of systems that have codified knowledge and learning systems to notice patterns in data (codifying new knowledge). |  | **✓** |  |
| A defined system which integrates inputted data points and uses a predictive model accordingly, limited by the programming. There is in fact no current AI which can pass a Turing test, thus it is a misnomer and misleading. That said, computer algorithms certainly have a place in diagnostic imaging and, to a much lesser extent, certain narrow aspects of diagnostic medicine in general, provided the limitations are inherently understood. |  | **✓** |  |
| Computer/technological algorithms that mimic problem solving abilities and thinking. |  | **✓** |  |
| A computerized algorithm that mimics cognition. | **✓** |  |  |
| A program that is meant to simulate thoughts and functions of a human. | **✓** |  |  |
| Computer or technology making decisions or completing tasks that would normally be done by a human. | **✓** |  |  |
| Computer learning algorithm, that is responsive to user inputs for calibration to generate increasingly accurate responses to a set of variables. |  | **✓** |  |
| AI a computer/web base service which help to triage/diagnose patients. |  |  | **✓** |
| A computer system help to triage, diagnose and manage patients. |  |  | **✓** |
| Autonomous machine learning. |  |  | **✓** |
| A computer algorithm that is designed to increase efficiency for the task at hand. |  |  | **✓** |
| Computer learning to derive answers/solutions based on previous data. |  |  | **✓** |
| Using algorithms to predict outcomes. |  |  | **✓** |
| Using a computer to do a task typically done by a human. |  | **✓** |  |
| Systems/machines/programs that can learn and improve themselves from information received. |  | **✓** |  |
| Computer-generated algorithms that attempt to predict real-world situations. | **✓** |  |  |
| The ability of a machine or software application to do tasks previously requiring human intelligence by using complex algorithms to learn, make decisions and problem solve. | **✓** |  |  |
| The use of computer algorithms and machine learning to analyse data. |  | **✓** |  |
| Tasks normally performed by humans are performed by computer systems programmed to perform the task and also to learn from performing the task. | **✓** |  |  |
| Computer program that can perform reasoning tasks usually done by human. | **✓** |  |  |
| Technology that aims to emulate human intelligence . | **✓** |  |  |
| Computer learning where data captured by the computer system helps to inform decision making going forward. |  | **✓** |  |
| The ability of a machine that has computer ability to use data and incorporate it to vary the response to a problem. |  |  | **✓** |
| Ways in which technology and computer software increase efficiency and take over tasks that overwise would have a human element involved. | **✓** |  |  |
| Technologies that have the capability to teach themselves new information e.g. 'I'm not a robot' - when you select which images in the grid contain a specific object. |  | **✓** |  |
| A machine capable of teaching, learning and executing tasks with rationale similar to that of humans. | **✓** |  |  |
| A model of iterative computer learning but constant inputting of new data. |  | **✓** |  |
| Software that can receive input and make decisions based on it within a defined system. Eg a program that can play chess against a competitor. |  | **✓** |  |
| The concept and development of utilizing computer systems to perform tasks that would otherwise require human intelligence. | **✓** |  |  |
| Computerized programs/algorithms designed to help reduce human error. |  |  | **✓** |
| Computerized /programmed algorithm to facilitate or take over human tasks. |  | **✓** |  |
| Technology aiding in decision making or every-day tasks. |  |  | **✓** |
| Technology to aid with decision making or processes that gathers input to deliver output. |  |  | **✓** |
| Having computers synthesize information and make decisions. |  | **✓** |  |
| Use of machines to solve problems. |  |  | **✓** |
| Using computer based dynamic algorithms to help in decision making process. |  | **✓** |  |
| Use of technology to make tasks easier. |  |  | **✓** |
| Intelligence of computers to be able to perform tasks normally completely by humans. | **✓** |  |  |
| Self-learning technologies that are able to collect & analyze large some of data, independently learn from that data, make deductions, and eventually make real-time decisions about related issues. | **✓** |  |  |
| Algorithms that 'learn' or adjust their responses based on incoming information, and which do tasks previously done by humans. | **✓** |  |  |
| Ability of a computer to do higher level tasks/decision making/problem solving. |  | **✓** |  |
| The ability to make sound decisions based on programming and data. |  | **✓** |  |
| The use of big data (for example routinely collected medical data) to predict outcomes, aid in medical decisions or automatically activate an action. |  | **✓** |  |
| Unsure |  |  | **✓** |
| A machine/computational system with capacity to carry out traditionally human-only tasks, i.e. emulating human intelligence. | **✓** |  |  |
| Machine learning based on data points in order to predict outcomes or recommend tests/outcomes |  | **✓** |  |
| A more sophisticated way of interpreting inputs than simple algorithmic approaches to provide outputs of greater accuracy. |  | **✓** |  |
| Machines and algorithms with the ability to learn, beyond simple pattern recognition. |  | **✓** |  |
| Technology that helps solve problems. |  |  | **✓** |
| A non-living system that can gain function beyond its explicit programming or instructions. |  | **✓** |  |
| Depending on the programming and input and object of the purpose, AI can be very useful to indicate decision outcomes and the synopsis of reasoning behind those decisions as well as the probability of accuracy of the recommendations. The strength of AI programs depends on the mega data input from related situations. |  | **✓** |  |
| Computers with the ability to make decisions based on information that is put into their database. |  | **✓** |  |
| The ability of a computer to process and learn from data in a manner consistent with human decision-making. | **✓** |  |  |
| Computers working to do processing of large data and 'make decisions'. |  | **✓** |  |
| Using and creating algorithms to make decisions. |  |  | **✓** |
| Computer generated information +/- machine learning. |  | **✓** |  |
| A computer program making decisions that a human being would otherwise be making. | **✓** |  |  |
| Machine based inductive learning based on deep neuronal systems. |  | **✓** |  |
| Computers learning to do things that humans do. |  | **✓** |  |
| The ability of computers/informatics to analyze or solve complex problems. |  | **✓** |  |
| An algorithm-based decision-making program which makes decisions based on information input. |  | **✓** |  |
| The development of algorithms and technological tools that can improve thought process and decision-making. |  |  | **✓** |
| Computer algorithm used to make human-like decisions. | **✓** |  |  |
| No idea. |  |  | **✓** |
| Using computer protocol to generate hypotheses based on inputted data. |  |  | **✓** |
| Programs able to do data analysis beyond applying simple rules and able to come to original results. |  | **✓** |  |
| The state of auto-adaptation of technology to tailor the users needs. |  | **✓** |  |
| The ability of a computer to interpret, understand, and act in a way that generally could only be done by humans. | **✓** |  |  |
| A computer algorithm that is capable of incorporating new information and learning, then using it to make decisions. |  | **✓** |  |
| Computer programs to assist a human in understanding and interpretation of information. |  |  | **✓** |
| Use of a machine, gadget, etc to help problem solve. |  |  | **✓** |
| Using technology to make decisions, understand the world. |  |  | **✓** |
| Using a computer algorithm to detect patterns. |  |  | **✓** |
| Machine based learning. |  | **✓** |  |
| The ability for a computer to perform a task that typically requires human judgment. Normally done via machine learning - use a large data set to train the computer on what something is and then it can use that 'experience' to make future decisions. | **✓** |  |  |
| The ability of technology to do basic human tasks |  |  | **✓** |
| Computer program that is designed to be self-adapting? |  | **✓** |  |
| Computer systems that can perform tasks that a person can do. |  | **✓** |  |
| Using technology to help/ease with our daily life activities . |  |  | **✓** |
| Computer/electronic based technology that is able to learn and adapt, solve problems. |  | **✓** |  |
| Machine learning and integration into practical work solutions |  | **✓** |  |
| The ability of a technical device to learn and be taught so as to interpret situations in a manner that replicates the human brain. | **✓** |  |  |
| Independent thought by a non sentient being. |  | **✓** |  |
| Computer driven decision making. |  |  | **✓** |
| The use of technology based algorithms to make decisions and/or perform human tasks. | **✓** |  |  |
| Intelligence of technology. |  |  | **✓** |
| Technology that is able to learn tasks (such as diagnosis) from initial parameters and outcome data. |  | **✓** |  |
| Databased or experience based computer learning that is ongoing and evolving. |  | **✓** |  |
| The ability of machines/computers to learn and develop beyond their initial programming |  | **✓** |  |
| In healthcare, technology-based tools designed to 'learn' for purposes of assessment, diagnosis, treatment. |  |  | **✓** |
| Utilisation de donnes pour faire des tâches de raisonnement normalement 'humaines'  (Using data to conduct (cognitive or reasoning) tasks that are typically “human”) | **✓** |  |  |
| Use of computers to solve problems.. |  |  | **✓** |
| A computer algorithm or process that can mimic or replicate human thought pattern or process. | **✓** |  |  |
| Aid to decisions via technology. |  |  | **✓** |
| Using computer technology for more important tasks. |  |  | **✓** |
| A non-organic intelligence that is able to reason and make logical deductions. | **✓** |  |  |
| Ability to use reasoning to come to decisions by non-living machine. | **✓** |  |  |
| The ability to use computers to analyze data and synthesize information into meaningful patterns which can be used to draw conclusions. |  |  | **✓** |
| Artificial intelligence is a computer system that can perform task commonly performed by humans. AI systems or neural network can learn from large data sets. | **✓** |  |  |
| Computer algorithms that process data and learn / solve problems. |  | **✓** |  |
| A primarily non-biological device or entity that can learn from experiences and adapt to its responses to its environment. |  | **✓** |  |
| Computers providing 'decisions' based on inputted data without using predetermined pathways. |  | **✓** |  |
| Problem solving/learning by computers/machines. |  | **✓** |  |
| The ability to integrate data and provide information about the meaning of the integrated information |  |  | **✓** |
| Computer generated protocols/programs that can adjust their output independently based on input data. |  |  | **✓** |
| When computers do things that humans would otherwise do. |  | **✓** |  |
| Systems that use computers and technology to perform tasks that otherwise have to be done by the human brain, |  |  |  |
| Unsure. |  |  | **✓** |
| Algorithms that change depending on the data that is collected. |  |  | **✓** |
| Ability of computer to learn. |  | **✓** |  |
| Computer software that can find associations and make connections between data that it was not directly programed to do. |  | **✓** |  |
| Using technology to aid or replace humans in the workplace. |  |  | **✓** |
| Developing, training, and using computational technology to assist in complex decision making tasks, analytics, or creation of novel constructs. |  | **✓** |  |
| Automated computer learning and application of it's knowledge. |  | **✓** |  |
| A field centred on the use of computer technology to performed typically by humans. | **✓** |  |  |
| Computer generated algorithms. |  |  | **✓** |
| Unsure |  |  | **✓** |
| Automation of certain thinking processes that can be done faster by a computer and more accurate than a human. |  | **✓** |  |
| The use of machines to mimic humans. |  | **✓** |  |
| Computer programming that allows an algorithm to arrive to a conclusion given a set of variables as input. |  |  | **✓** |
| Problem solving by a computer. |  |  | **✓** |
| Computer algorithms that allow for complex questions, tasks or interactions to be managed by computers. |  |  | **✓** |
| Computers ability to recognize patterns and make decisions based on them. |  |  | **✓** |
| Technology that allows computers to simulate human thinking and perform tasks in real-world context. | **✓** |  |  |
| Computers solving problems for us. |  |  | **✓** |
| When an artificial entity is able to make decisions. |  | **✓** |  |
| Computers to predict and gain information. |  |  | **✓** |
| Non-human capable of making decisions based on series of algorithms. |  |  | **✓** |
| Machine learning that aids in task completion. |  | **✓** |  |
| The ability of a biological entity to integrate and process information for decision making. |  | **✓** |  |
| Computer that does the function of a person. |  | **✓** |  |
| Machine/ software tools that allow computers to provide solutions to problems usually requiring human thought. | **✓** |  |  |
| A system based technology to utilize human problem solving ability. |  | **✓** |  |
| Computers or other technology to help with problem solving. |  |  | **✓** |
| Using computers to make decisions that humans usually would. |  | **✓** |  |
| Technology that supports decision making and treatment intervention. |  |  | **✓** |
| Using technology to do simple mundane repetitive tasks based on an algorithm. |  |  | **✓** |
| Automated iterative learning computational process. |  | **✓** |  |
| Intelligence demonstrated by machines (computers or similar technology), which may- but usually does not- resemble human intelligence. | **✓** |  |  |
| Any form of informatics system (like an EMR) that use technology and not humans/ paper |  |  | **✓** |
| The ability of a machine to do something 'intelligent', i.e. like a human would do that involves synthesizing information and generating a conclusion. | **✓** |  |  |
| Autonomous decisions made by software that require little human input and control. |  | **✓** |  |
| The ability of a computer to do work (physical an mental ) normally done by humans. |  | **✓** |  |
| Computer based technology that can make decisions based on inputted or absorbed information. |  |  | **✓** |
| The use of statistics and mathematics as applied to observational studies to developed a course of action. |  |  | **✓** |
| Computer algorithms used to make decisions. |  |  | **✓** |
| A self-learning algorithm capable of making its own complex decisions. The decision-making process may be obscure to its creator. |  | **✓** |  |
| What we call 'AI' in medicine is often more specifically machine learning (typically with adversarial or convolutional neural networks). General AI (that is, intelligence demonstrated by machines that roughly replicates human intelligence) is very far off in the future. | **✓** |  |  |
| The ability of a computer or a robot controlled by a computer to do tasks that are usually done by humans because they require human intelligence and discernment | **✓** |  |  |
| **5 ATTEMPTS LEFT BLANK |  |  | **(5) ✓** |
