# Supplementary material for: Needs and expectations for artificial intelligence in emergency medicine according to Canadian physicians
Source: BMC Health Serv Res. 2023 Jul 25;23:798. doi: 10.1186/s12913-023-09740-w (PMC10369807; doi:10.1186/s12913-023-09740-w)
Supplement: Supplementary file 4 — Additional file 4. Appendix D: Supplemental Results. [file 12913_2023_9740_MOESM4_ESM.docx]

**Appendix D: Supplemental Results**

| **Table 2: Additional Physician Demographic Data**​ | | | |
| --- | --- | --- | --- |
| ​ | ​ | ​ | ​ |
| ​ | ​ | **Total**​ | **Percent**​ |
| Time in Practice (n = 222)​ | ​ | ​ | ​ |
| Under 1 year​ | ​ | 1​ | 0.5%​ |
| 1 to 3 years​ | ​ | 42​ | 18.9%​ |
| 3 to 5 years​ | ​ | 21​ | 9.5%​ |
| 5 to 7 years​ | ​ | 23​ | 10.4%​ |
| 7 to 9 years​ | ​ | 17​ | 7.7%​ |
| 9 to 11 years​ | ​ | 15​ | 6.8%​ |
| 11 to 13 years​ | ​ | 7​ | 3.2%​ |
| 13 to 15 years​ | ​ | 9​ | 4.1%​ |
| 15 to 17 years​ | ​ | 9​ | 4.1%​ |
| 17 to 19 years​ | ​ | 13​ | 5.9%​ |
| 19 to 21 years​ | ​ | 11​ | 5.0%​ |
| 21 to 23 years​ | ​ | 8​ | 3.6%​ |
| 23 to 25 years​ | ​ | 6​ | 2.7%​ |
| 25 to 27 years​ | ​ | 6​ | 2.7%​ |
| 27 to 29 years​ | ​ | 6​ | 2.7%​ |
| 29 to 31 years​ | ​ | 7​ | 3.2%​ |
| 31 to 33 years​ | ​ | 1​ | 0.5%​ |
| 33 to 35 years​ | ​ | 3​ | 1.4%​ |
| 35 years or more​ | ​ | 17​ | 7.7%​ |
|  |  |  |  |
| Employment Status (n = 230)​ | ​ | ​ | ​ |
| Full Time​ | ​ | 170​ | 73.9%​ |
| Part Time​ | ​ | 39​ | 17.0%​ |
| Not Working​ | ​ | 21​ | 9.1%​ |

| **Table 3: Priority Ranking for Translation of Future AI Tools in Clinical Practice**​ | | | | | | | | | |
| --- | --- | --- | --- | --- | --- | --- | --- | --- | --- |
| ​ | ​ |  | | | | | | ​ | ​ |
| ​ | ​ | **Frequency of Priority Ranking**​ | | | | | | ​ | **Total Ranking by Weighted Points**​ |
| ​ | ​ | **1st Priority**​ | | **2nd Priority**​ | | **Third Priority**​ | | ​ |  |
| ​ | ​ | ​ | ​ | ​ | ​ | ​ | ​ | ​ | ​ |
| **Examples of AI Applications by ED Physician Work-Types (n = 186)**​ | ​ | ​ | ​ | ​ | ​ | ​ | ​ | ​ | ​ |
| AI tools for documentation​ | ​ | 61​ | (32.8%)​ | 41​ | (22.0%)​ | 21​ | (11.3%)​ | ​ | 286​ |
| AI tools for computer Use​ | ​ | 29​ | (15.6%)​ | 58​ | (31.2%)​ | 35​ | (18.8%)​ | ​ | 238​ |
| Triaging of patients​ | ​ | 40​ | (21.5%)​ | 26​ | (14.0%)​ | 33​ | (17.7%)​ | ​ | 205​ |
| AI tools to diagnose, select investigations, risk stratify​ | ​ | 26​ | (14.0%)​ | 24​ | (12.9%)​ | 18​ | (9.7%)​ | ​ | 144​ |
| AI tools for Administration, Education & Research​ | ​ | 6​ | (3.2%)​ | 12​ | (6.5%)​ | 27​ | (14.5%)​ | ​ | 69​ |
| AI tools for History Taking​ | ​ | 11​ | (5.9%)​ | 11​ | (5.9%)​ | 11​ | (5.9%)​ | ​ | 66​ |
| AI tools for Communication​ | ​ | 4​ | (2.2%)​ | 7​ | (3.8%)​ | 22​ | (11.8%)​ | ​ | 48​ |
| AI tools for Physical Exam & Bedside procedures​ | ​ | 4​ | (2.2%)​ | 6​ | (3.2%)​ | 11​ | (5.9%)​ | ​ | 35​ |
| Other​ | ​ | 4​ | (2.2%)​ | 1​ | (0.5%)​ | 5​ | (2.7%)​ | ​ | 19​ |
| AI tools for Teaching and Supervision​ | ​ | 1​ | (0.5%)​ | 0​ | (0.0%)​ | 3​ | (1.6%)​ | ​ | 6​ |
| ​ | ​ | ​ | ​ | ​ | ​ | ​ | ​ | ​ | ​ |
| **Examples of Published AI Applications for ED Patient Care (n = 177)**​ | ​ | ​ | ​ | ​ | ​ | ​ | ​ | ​ | ​ |
| Automated ED charting or report generation​ | ​ | 50​ | (28.2%)​ | 18​ | (10.2%)​ | 20​ | (11.3%)​ | ​ | 206​ |
| Clinical Prediction Rules​ | ​ | 17​ | (9.6%)​ | 37​ | (20.9%)​ | 11​ | (6.2%)​ | ​ | 136​ |
| Monitoring of vitals in ED with early warning detection​ | ​ | 23​ | (13.0%)​ | 23​ | (13.0%)​ | 12​ | (6.8%)​ | ​ | 127​ |
| Predicting ED demand & workload to aid in scheduling human resources​ | ​ | 14​ | (7.9%)​ | 25​ | (14.1%)​ | 23​ | (13.0%)​ | ​ | 115​ |
| Imaging interpretation​ | ​ | 17​ | (9.6%)​ | 16​ | (9.0%)​ | 22​ | (12.4%)​ | ​ | 105​ |
| Predicting diagnosis & recommending treatment for common diseases​ | ​ | 19​ | (10.7%)​ | 7​ | (4.0%)​ | 12​ | (6.8%)​ | ​ | 83​ |
| Precision medication ordering / dosing​ | ​ | 7​ | (4.0%)​ | 13​ | (7.3%)​ | 17​ | (9.6%)​ | ​ | 64​ |
| Assessing discharge notes to predict readmission and returns to ED​ | ​ | 7​ | (4.0%)​ | 5​ | (2.8%)​ | 9​ | (5.1%)​ | ​ | 40​ |
| Recommendation of handouts and patient resources​ | ​ | 1​ | (0.6%)​ | 10​ | (5.6%)​ | 17​ | (9.6%)​ | ​ | 40​ |
| Performing or interpreting POCUS​ | ​ | 5​ | (2.8%)​ | 9​ | (5.1%)​ | 5​ | (2.8%)​ | ​ | 38​ |
| Triage or disposition prediction​ | ​ | 7​ | (4.0%)​ | 3​ | (1.7%)​ | 10​ | (5.6%)​ | ​ | 37​ |
| Analyzing patient experiences using social media to inform QI initiatives​ | ​ | 3​ | (1.7%)​ | 2​ | (1.1%)​ | 6​ | (3.4%)​ | ​ | 19​ |
| Prehospital Patient Monitoring​ | ​ | 4​ | (2.3%)​ | 0​ | (0.0%)​ | 4​ | (2.3%)​ | ​ | 16​ |
| Training systems for advanced procedures​ | ​ | 1​ | (0.6%)​ | 5​ | (2.8%)​ | 3​ | (1.7%)​ | ​ | 16​ |
| Screening patients for clinical trial eligibility or aiding research​ | ​ | 0​ | (0.0%)​ | 2​ | (1.1%)​ | 6​ | (3.4%)​ | ​ | 10​ |
| Other​ | ​ | 2​ | (1.1%)​ | 2​ | (1.1%)​ | 0​ | (0.0%)​ | ​ | 10​ |

| **Table 4: Prior Education and Technology Experience**​ | | | | | |
| --- | --- | --- | --- | --- | --- |
|  | ​ | ​ | ​ | ​ |  |
|  | ​ | ​ | **Total**​ | **Percent**​ |  |
|  | *Prior Education Experience (n=212)*​ | ​ | ​ | ​ |  |
|  | Arts​ | ​ | 18​ | 6.7%​ |  |
|  | Computer Science​ | ​ | 6​ | 2.2%​ |  |
|  | Commerce​ | ​ | 8​ | 3.0%​ |  |
|  | Engineering​ | ​ | 12​ | 4.4%​ |  |
|  | Life Sciences​ | ​ | 157​ | 58.1%​ |  |
|  | Physical Sciences​ | ​ | 39​ | 14.4%​ |  |
|  | Social Sciences​ | ​ | 8​ | 3.0%​ |  |
|  | Other Please Specify​ | ​ | 22​ | 8.1%​ |  |
|  | ​ | ​ | ​ | ​ |  |
|  | *Self-Rated Interest in Technology (n=212)*​ | ​ | ​ | ​ |  |
|  | I am a Technology Enthusiast​ | ​ | 70​ | 33.0%​ |  |
|  | I am a Technology Hobbiest​ | ​ | 43​ | 20.3%​ |  |
|  | I sudied computer technology​ | ​ | 16​ | 7.5%​ |  |
|  | I studied information technology​ | ​ | 12​ | 5.7%​ |  |
|  | I have no interest in technology​ | ​ | 19​ | 9.0%​ |  |
|  | I neither like nor dislike technology​ | ​ | 91​ | 42.9%​ |  |

| **Table 4 (Continued): Prior Education and Technology Experience**​ | | | | | | | | | | | |  |  |
| --- | --- | --- | --- | --- | --- | --- | --- | --- | --- | --- | --- | --- | --- |
| ​ | ​ | ​ | ​ | ​ | ​ | | ​ | | ​ | | | | |
| ​ | ​ | ​ | **Total**​ | **Percent**​ | ​ | | **Measure of Central Tendency**​ | | | | |  |  |
| ​ | **TechPH Scale (n = 212)**​ | ​ | ​ | ​ | ​ | | ​ | ​ | | | | |  |
| ​ | *Technology Enthusiasm*​ | ​ | ​ | ​ | ​ | | ​ | ​ | | | | |  |
| ​ | I think new technological gadgets are fun​ | ​ | ​ | ​ | ​ | | ​ | | | ​ | |  |  |
| ​ | Strongly Disagree​ | ​ | 1​ | 0.5%​ | ​ | | **Mean**​ | | | 3.84​ | |  |  |
| ​ | Disagree​ | ​ | 12​ | 5.7%​ | ​ | | **Median**​ | | | 4​ | |  |  |
| ​ | Neutral​ | ​ | 45​ | 21.2%​ | ​ | | **Mode**​ | | | 4​ | |  |  |
| ​ | Agree​ | ​ | 116​ | 54.7%​ | ​ | | **Interpretation**​ | | | (Agree)​ | |  |  |
| ​ | Strongly Agree​ | ​ | 38​ | 17.9%​ | ​ | | ​ | | | ​ | |  |  |
| ​ | ​ | ​ | ​ | ​ | ​ | | ​ | | | ​ | |  |  |
| ​ | Using technology makes life easier for me​ | ​ | ​ | ​ | ​ | | ​ | | | ​ | |  |  |
| ​ | Strongly Disagree​ | ​ | 2​ | 0.9%​ | ​ | | **Mean**​ | | | 3.98​ | |  |  |
| ​ | Disagree​ | ​ | 8​ | 3.8%​ | ​ | | **Median**​ | | | 4​ | |  |  |
| ​ | Neutral​ | ​ | 34​ | 16.0%​ | ​ | | **Mode**​ | | | 4​ | |  |  |
| ​ | Agree​ | ​ | 117​ | 55.2%​ | ​ | | **Interpretation**​ | | | (Agree)​ | |  |  |
| ​ | Strongly Agree​ | ​ | 51​ | 24.1%​ | ​ | | ​ | | | ​ | |  |  |
| ​ | ​ | ​ | ​ | ​ | ​ | | ​ | | | ​ | |  |  |
| ​ | I like to acquire the latest models and updates​ | ​ | ​ | ​ | ​ | | ​ | | | ​ | |  |  |
| ​ | Strongly Disagree​ | ​ | 10​ | 4.7%​ | ​ | | **Mean**​ | | | 3.19​ | |  |  |
| ​ | Disagree​ | ​ | 44​ | 20.8%​ | ​ | | **Median**​ | | | 3​ | |  |  |
| ​ | Neutral​ | ​ | 74​ | 34.9%​ | ​ | | **Mode**​ | | | 3​ | |  |  |
| ​ | Agree​ | ​ | 63​ | 29.7%​ | ​ | | **Interpretation**​ | | | (Neutral)​ | |  |  |
| ​ | Strongly Agree​ | ​ | 21​ | 9.9%​ | ​ | | ​ | | | ​ | |  |  |
| ​ | ​ | | ​ | ​ | ​ | ​ | ​ | | | |  |  |  |
| ​ | I understand what is meant by artificial intelligence​ | | ​ | ​ | ​ | ​ | ​ | | | |  |  |  |
| ​ | Strongly Disagree​ | ​ | 1​ | 0.5%​ | ​ | | **Mean**​ | | | 4.10​ | |  |  |
| ​ | Disagree​ | ​ | 2​ | 0.9%​ | ​ | | **Median**​ | | | 4​ | |  |  |
| ​ | Neutral​ | ​ | 24​ | 11.3%​ | ​ | | **Mode**​ | | | 4​ | |  |  |
| ​ | Agree​ | ​ | 133​ | 62.7%​ | ​ | | **Interpretation**​ | | | (Agree)​ | |  |  |
| ​ | Strongly Agree​ | ​ | 52​ | 24.5%​ | ​ | | ​ | | | ​ | |  |  |
| ​ |  | ​ | ​ | ​ | ​ | | ​ | | | ​ | |  |  |

| **Table 4 (Continued): Prior Education and Technology Experience**​ | | | | | | | | | | | | | | | | |  |
| --- | --- | --- | --- | --- | --- | --- | --- | --- | --- | --- | --- | --- | --- | --- | --- | --- | --- |
| ​ | | ​ | | | ​ | ​ | | ​ | | | ​ | ​ | | ​ | | | |
| ​ | | ​ | | | ​ | **Total**​ | | **Percent**​ | | | ​ | **Measure of Central Tendency**​ | | | | | |
| ​ | | **TechPH Scale (N = 212)**​ | | | ​ | ​ | | ​ | | | ​ | ​ | | ​ | | | |
| ​ | | *Technology Anxiety*​ | | | ​ | ​ | | ​ | | | ​ | ​ | | ​ | | | |
| ​ | | I am sometimes afraid of not being able to use new technological things​ | | | ​ | ​ | | ​ | | | ​ | ​ | | ​ | | | |
| ​ | | Strongly Disagree​ | | | ​ | 25​ | | 11.8%​ | | | ​ | **Mean**​ | | 2.80​ | | | |
| ​ | | Disagree​ | | | ​ | 71​ | | 33.5%​ | | | ​ | **Median**​ | | 3​ | | | |
| ​ | | Neutral​ | | | ​ | 47​ | | 22.2%​ | | | ​ | **Mode**​ | | 2​ | | | |
| ​ | | Agree​ | | | ​ | 58​ | | 27.4%​ | | | ​ | **Interpretation**​ | | (DisAg / Neut)​ | | | |
| ​ | | Strongly Agree​ | | | ​ | 11​ | | 5.2%​ | | | ​ | ​ | | ​ | | | |
| ​ | | ​ | | | ​ | ​ | | ​ | | | ​ | ​ | | ​ | | | |
| ​ | | Today technological progress is so fast, its hard to keep up​ | | | ​ | ​ | | ​ | | | ​ | ​ | | ​ | | | |
| ​ | | Strongly Disagree​ | | | ​ | 9​ | | 4.2%​ | | | ​ | **Mean**​ | | 3.35​ | | | |
| ​ | | Disagree​ | | | ​ | 47​ | | 22.2%​ | | | ​ | **Median**​ | | 4​ | | | |
| ​ | | Neutral​ | | | ​ | 42​ | | 19.8%​ | | | ​ | **Mode**​ | | 4​ | | | |
| ​ | | Agree​ | | | ​ | 88​ | | 41.5%​ | | | ​ | **Interpretation**​ | | (DisAg / Neut)​ | | | |
| ​ | | Strongly Agree​ | | | ​ | 26​ | | 12.3%​ | | | ​ | ​ | | ​ | | | |
| ​ | | ​ | | | ​ | ​ | | ​ | | | ​ | ​ | | ​ | | | |
| ​ | | I would have dared to try new technological things if I had more help​ | | | ​ | ​ | | ​ | | | ​ | ​ | | ​ | | | |
| ​ | | Strongly Disagree​ | | | ​ | 14​ | | 6.6%​ | | | ​ | **Mean**​ | | 3.04​ | | | |
| ​ | | Disagree​ | | | ​ | 54​ | | 25.5%​ | | | ​ | **Median**​ | | 3​ | | | |
| ​ | | Neutral​ | | | ​ | 63​ | | 29.7%​ | | | ​ | **Mode**​ | | 4​ | | | |
| ​ | | Agree​ | | | ​ | 71​ | | 33.5%​ | | | ​ | **Interpretation**​ | | (Neutral)​ | | | |
| ​ | | Strongly Agree​ | | | ​ | 10​ | | 4.7%​ | | | ​ | ​ | | ​ | | | |
| ​ | | ​ | | | ​ | ​ | | ​ | | | ​ | ​ | | ​ | | | |
| ​ | | **Overall TechPH Score**​ | | | ​ | ​ | | ​ | | | ​ | **Mean**​ | | 3.30​ | | | |
| ​ | | ​ | | | ​ | ​ | | ​ | | | ​ | **St Dev**​ | | 0.650​ | | | |
|  | |  | | |  |  | |  | | |  |  | |  | | | |
| **Table 5: Prior Experience with AI**​ | | | | | | | | | | | | | | |  |  |  |
| ​ | ​ | | ​ | ​ | | | ​ | | ​ | ​ | | | ​ | | |  |  |
| ​ | ​ | | ​ | **Total**​ | | | **Percent**​ | | ​ | ​ | | | ​ | | |  |  |
| ​ | **Academic Experience with AI (n = 199)**​ | | ​ | ​ | | | ​ | | ​ | ​ | | | ​ | | |  |  |
| ​ | I have read journal articles on AI in general​ | | ​ | 76​ | | | 38.2%​ | | ​ | ​ | | | ​ | | |  |  |
| ​ | I have read journal articles on AI in medicine​ | | ​ | 90​ | | | 45.2%​ | | ​ | ​ | | | ​ | | |  |  |
| ​ | I have conducted research on AI in general​ | | ​ | 3​ | | | 1.5%​ | | ​ | ​ | | | ​ | | |  |  |
| ​ | I have conducted research on AI in medicine​ | | ​ | 8​ | | | 4.0%​ | | ​ | ​ | | | ​ | | |  |  |
| ​ | None of the above​ | | ​ | 77​ | | | 38.7%​ | | ​ | ​ | | | ​ | | |  |  |
| ​ | Other​ | | ​ | 5​ | | | 2.5%​ | | ​ | ​ | | | ​ | | |  |  |
| ​ | ​ | | ​ | ​ | | | ​ | | ​ | ​ | | | ​ | | |  |  |
| ​ | **Personal Experience with AI (n = 199)**​ | | ​ | ​ | | | ​ | | ​ | ​ | | | ​ | | |  |  |
| ​ | In my Personal Life​ | | ​ | ​ | | | ​ | | ​ | ​ | | | ​ | | |  |  |
| ​ | I don't know​ | | ​ | 6​ | | | 3.0%​ | | ​ | ​ | | | ​ | | |  |  |
| ​ | None at all​ | | ​ | 30​ | | | 15.1%​ | | ​ | **Mean**​ | | | 3.51​ | | |  |  |
| ​ | Very Little​ | | ​ | 55​ | | | 27.6%​ | | ​ | **Median**​ | | | 4​ | | |  |  |
| ​ | Some​ | | ​ | 76​ | | | 38.2%​ | | ​ | **Mode**​ | | | 4​ | | |  |  |
| ​ | Quite a Bit​ | | ​ | 28​ | | | 14.1%​ | | ​ | **Interpretation**​ | | | (Very Little - Some)​ | | |  |  |
| ​ | A great deal​ | | ​ | 4​ | | | 2.0%​ | | ​ | ​ | | | ​ | | |  |  |
| ​ | ​ | | ​ | ​ | | | ​ | | ​ | ​ | | | ​ | | |  |  |
| ​ | *In my Clinical Work in General*​ | | ​ | ​ | | | ​ | | ​ | ​ | | | ​ | | |  |  |
| ​ | I don't know​ | | ​ | 6​ | | | 3.0%​ | | ​ | ​ | | | ​ | | |  |  |
| ​ | None at all​ | | ​ | 73​ | | | 36.7%​ | | ​ | **Mean**​ | | | 2.91​ | | |  |  |
| ​ | Very Little​ | | ​ | 63​ | | | 31.7%​ | | ​ | **Median**​ | | | 3​ | | |  |  |
| ​ | Some​ | | ​ | 48​ | | | 24.1%​ | | ​ | **Mode**​ | | | 2​ | | |  |  |
| ​ | Quite a Bit​ | | ​ | 8​ | | | 4.0%​ | | ​ | **Interpretation**​ | | | (Very Little)​ | | |  |  |
| ​ | A great deal​ | | ​ | 1​ | | | 0.5%​ | | ​ | ​ | | | ​ | | |  |  |
| ​ | ​ | | ​ | ​ | | | ​ | | ​ | ​ | | | ​ | | |  |  |
| ​ | *In my work as an Emergency Physician*​ | | ​ | ​ | | | ​ | | ​ | ​ | | | ​ | | |  |  |
| ​ | I don't know​ | | ​ | 9​ | | | 4.5%​ | | ​ | ​ | | | ​ | | |  |  |
| ​ | None at all​ | | ​ | 87​ | | | 43.7%​ | | ​ | **Mean**​ | | | 2.77​ | | |  |  |
| ​ | Very Little​ | | ​ | 54​ | | | 27.1%​ | | ​ | **Median**​ | | | 3​ | | |  |  |
| ​ | Some​ | | ​ | 41​ | | | 20.6%​ | | ​ | **Mode**​ | | | 2​ | | |  |  |
| ​ | Quite a Bit​ | | ​ | 6​ | | | 3.0%​ | | ​ | **Interpretation**​ | | | (Very Little)​ | | |  |  |
| ​ | A great deal​ | | ​ | 2​ | | | 1.0%​ | | ​ | ​ | | | ​ | | |  |  |
| ​ | ​ | | ​ | ​ | | | ​ | | ​ | ​ | | | ​ | | |  |  |
| ​ | *In Research*​ | | ​ | ​ | | | ​ | | ​ | ​ | | | ​ | | |  |  |
| ​ | I don't know​ | | ​ | 7​ | | | 3.5%​ | | ​ | ​ | | | ​ | | |  |  |
| ​ | None at all​ | | ​ | 142​ | | | 71.4%​ | | ​ | **Mean**​ | | | 2.41​ | | |  |  |
| ​ | Very Little​ | | ​ | 22​ | | | 11.1%​ | | ​ | **Median**​ | | | 2​ | | |  |  |
| ​ | Some​ | | ​ | 21​ | | | 10.6%​ | | ​ | **Mode**​ | | | 2​ | | |  |  |
| ​ | Quite a Bit​ | | ​ | 4​ | | | 2.0%​ | | ​ | **Interpretation**​ | | | (None - Very Little)​ | | |  |  |
| ​ | A great deal​ | | ​ | 3​ | | | 1.5%​ | | ​ | ​ | | | ​ | | |  |  |
| ​ | ​ | | ​ | ​ | | | ​ | | ​ | ​ | | | ​ | | |  |  |

| **Table 6: Prior Experience with AI**​ | | | | | | | |
| --- | --- | --- | --- | --- | --- | --- | --- |
|  | | | | | | | |
| ​ | ​ | **Used**​ | | **Heard Of**​ | | **Not Applicable**​ | |
| ​ | ​ | ​ | ​ | ​ | ​ |  | ​ |
| **Examples of AI Applications by EP Work-Activities (n = 189)**​ | ​ |  |  |  |  |  | ​ |
| AI tools for computer use​ | ​ | 55​ | (29.1%)​ | 76​ | (40.2%)​ | 58​ | (30.7%)​ |
| AI tools for documentation​ | ​ | 38​ | (20.1%)​ | 70​ | (37.0%)​ | 81​ | (42.9%)​ |
| AI tools for administration, education & research​ | ​ | 32​ | (16.9%)​ | 59​ | (31.2%)​ | 98​ | (51.9%)​ |
| AI tools for diagnosis, selecting investigations, risk stratification​ | ​ | 30​ | (15.9%)​ | 81​ | (42.9%)​ | 78​ | (41.3%)​ |
| AI tools for communication​ | ​ | 28​ | (14.8%)​ | 53​ | (28.0%)​ | 108​ | (57.1%)​ |
| Triaging of patients​ | ​ | 22​ | (11.6%)​ | 81​ | (42.9%)​ | 86​ | (45.5%)​ |
| AI tools for physical exam & bedside procedures​ | ​ | 21​ | (11.1%)​ | 42​ | (22.2%)​ | 126​ | (66.7%)​ |
| AI tools for teaching and supervision​ | ​ | 17​ | (9.0%)​ | 42​ | (22.2%)​ | 130​ | (68.8%)​ |
| AI tools for history taking​ | ​ | 9​ | (4.8%)​ | 52​ | (27.5%)​ | 128​ | (67.7%)​ |
| ​ | ​ | ​ | ​ | ​ | ​ | ​ | ​ |
| **Examples of Published AI Tools for ED Patient Care (n = 197)**​ | ​ | ​ | ​ | ​ | ​ | ​ | ​ |
| Clinical prediction rules​ | ​ | 102​ | (51.8%)​ | 73​ | (37.1%)​ | 22​ | (11.2%)​ |
| Monitoring of vitals in ED with early warning detection​ | ​ | 59​ | (29.9%)​ | 80​ | (40.6%)​ | 58​ | (29.4%)​ |
| Performing or interpreting POCUS​ | ​ | 50​ | (25.4%)​ | 70​ | (35.5%)​ | 77​ | (39.1%)​ |
| Recommendation of handouts and patient resources​ | ​ | 41​ | (20.8%)​ | 54​ | (27.4%)​ | 102​ | (51.8%)​ |
| Precision medication ordering / dosing​ | ​ | 37​ | (18.8%)​ | 74​ | (37.6%)​ | 86​ | (43.7%)​ |
| Automated ED charting or report generation​ | ​ | 26​ | (13.2%)​ | 81​ | (41.1%)​ | 90​ | (45.7%)​ |
| Predicting ED demand & workload to schedule human resources​ | ​ | 24​ | (12.2%)​ | 107​ | (54.3%)​ | 66​ | (33.5%)​ |
| Triage or disposition prediction​ | ​ | 20​ | (10.2%)​ | 70​ | (35.5%)​ | 107​ | (54.3%)​ |
| Diagnosing & recommending treatment for common diseases​ | ​ | 19​ | (9.6%)​ | 100​ | (50.8%)​ | 78​ | (39.6%)​ |
| Screening patients for clinical trial eligibility or aiding research​ | ​ | 17​ | (8.6%)​ | 60​ | (30.5%)​ | 120​ | (60.9%)​ |
| Training systems for advanced procedures​ | ​ | 15​ | (7.6%)​ | 69​ | (35.0%)​ | 113​ | (57.4%)​ |
| Imaging XRAY interpretation​ | ​ | 14​ | (7.1%)​ | 126​ | (64.0%)​ | 57​ | (28.9%)​ |
| Prehospital Patient Monitoring​ | ​ | 10​ | (5.1%)​ | 67​ | (34.0%)​ | 120​ | (60.9%)​ |
| Imaging CT Interpretation​ | ​ | 9​ | (4.6%)​ | 120​ | (60.9%)​ | 68​ | (34.5%)​ |
| Analyzing patient experiences with social media to inform QI initiatives​ | ​ | 7​ | (3.6%)​ | 76​ | (38.6%)​ | 114​ | (57.9%)​ |
| Imaging MRI interpretation​ | ​ | 5​ | (2.5%)​ | 110​ | (55.8%)​ | 82​ | (41.6%)​ |
| Imaging Formal US interpretation​ | ​ | 5​ | (2.5%)​ | 107​ | (54.3%)​ | 85​ | (43.1%)​ |
| Assessing discharge notes to predict readmission & returns to ED​ | ​ | 3​ | (1.5%)​ | 62​ | (31.5%)​ | 132​ | (67.0%)​ |
| ​ | | | | | | | |

|  | ​ | | ​ | ​ | | ​ | | ​ | ​ | | ​ | | |
| --- | --- | --- | --- | --- | --- | --- | --- | --- | --- | --- | --- | --- | --- |
| **Table 7: Perception of AI Tools for Emergency Medicine** | | | | | | | | | | | | |  |
|  | |  | | |  |  |  | | |  | |  |  |
|  | |  | | |  | **Total** | **Percent** | | |  | |  |  |
|  | | **Opinion on Potential for use of AI in ED (n =174)** | | |  |  |  | | |  | |  |  |
|  | | Not Applicable | | |  | 0 | 0.0% | | |  | |  |  |
|  | | No Potential | | |  | 0 | 0.0% | | |  | |  |  |
|  | | Very Little Potential | | |  | 5 | 2.9% | | |  | |  |  |
|  | | Some Potential | | |  | 43 | 24.7% | | |  | |  |  |
|  | | Quite a Bit of Potential | | |  | 69 | 39.7% | | |  | |  |  |
|  | | A Great deal of potential | | |  | 57 | 32.8% | | |  | |  |  |
|  | |  | | |  |  |  | | |  | |  |  |
|  | | **I am interested in the use of AI in EM (n = 171)** | | |  |  |  | | |  | |  |  |
|  | | Not Applicable | | |  | 0 | 0.0% | | |  | |  |  |
|  | | Strongly Disagree | | |  | 2 | 1.2% | | |  | |  |  |
|  | | Somewhat Disagree | | |  | 0 | 0.0% | | |  | |  |  |
|  | | Neutral | | |  | 18 | 10.5% | | |  | |  |  |
|  | | Somewhat Agree | | |  | 68 | 39.8% | | |  | |  |  |
|  | | Strongly Agree | | |  | 83 | 48.5% | | |  | |  |  |
|  | |  | | |  |  |  | | |  | |  |  |
|  | | **Impact of AI on Emergency Physicians over the next 10 years (n = 170)** | | | | | | | | | | |  |
|  | | Not Applicable | | |  | 0 | 0.0% | | |  | |  |  |
|  | | No Impact (Jobs will remain unchanged) | | |  | 6 | 3.5% | | |  | |  |  |
|  | | Minimal Impact (jobs will change slightly) | | |  | 103 | 60.6% | | |  | |  |  |
|  | | Moderate Impact (jobs will change substantially) | | |  | 61 | 35.9% | | |  | |  |  |
|  | | Extreme Impact (Jobs will become obsolete) | | |  | 0 | 0.0% | | |  | |  |  |
|  | |  | | |  |  |  | | |  | |  |  |
|  | | **Ranking of AI’s Ability to Complete the Following ED Physician Tasks:** | | | | | | | | | |  |  |
|  | | *Analyze patient information to reach a diagnosis (n = 174)* | | |  |  |  | | |  | |  |  |
|  | | Extremely Unlikely (1) | | |  | 17 | 9.8% | | | **Mean:** | | 3.53 |  |
|  | | Unlikely (2) | | |  | 50 | 28.7% | | | **Median:** | | 4 |  |
|  | | Neutral (3) | | |  | 43 | 24.7% | | | **Mode:** | | 4 |  |
|  | | Likely (4) | | |  | 51 | 29.3% | | | **Interpretation:** | | Neutral - Likely |  |
|  | | Extremely Likely (5) | | |  | 13 | 7.5% | | |  | |  |  |
|  | |  | | |  |  |  | | |  | |  |  |
|  | | *Analyze patient information to reach a prognosis (n = 174)* | | |  |  |  | | |  | |  |  |
|  | | Extremely Unlikely (1) | | |  | 6 | 3.4% | | | **Mean:** | | 3.25 |  |
|  | | Unlikely (2) | | |  | 19 | 10.9% | | | **Median:** | | 3 |  |
|  | | Neutral (3) | | |  | 48 | 27.6% | | | **Mode:** | | 4 |  |
|  | | Likely (4) | | |  | 78 | 44.8% | | | **Interpretation:** | | Neutral - Likely |  |
|  | | Extremely Likely (5) | | |  | 23 | 13.2% | | |  | |  |  |

| **Table 7 (Continued): Perception of AI Tools for Emergency Medicine** | | | | |  |  |
| --- | --- | --- | --- | --- | --- | --- |
|  |  |  |  |  |  |  |
|  |  |  | **Total** | **Percent** |  |  |
|  | **Ranking of AI’s Ability to Complete the Following ED Physician Tasks:** |  |  |  |  |  |
|  |  |  |  |  |  |  |
|  | *Formulate personalized treatment plans (n = 174)* |  |  |  |  |  |
|  | Extremely Unlikely |  | 9 | 5.2% | **Mean:** | 3.25 |
|  | Unlikely |  | 28 | 16.1% | **Median:** | 3 |
|  | Neutral |  | 60 | 34.5% | **Mode:** | 4 |
|  | Likely |  | 64 | 36.8% | **Interpretation:** | Neutral – Likely |
|  | Extremely Likely |  | 13 | 7.5% |  |  |
|  |  |  |  |  |  |  |
|  | Provide documentation *(n = 174)* |  |  |  |  |  |
|  | Extremely Unlikely |  | 1 | 0.6% | **Mean:** | 4.27 |
|  | Unlikely |  | 4 | 2.3% | **Median:** | 4 |
|  | Neutral |  | 18 | 10.3% | **Mode:** | 5 |
|  | Likely |  | 75 | 43.1% | **Interpretation:** | Extremely Likely |
|  | Extremely Likely |  | 76 | 43.7% |  |  |
|  |  |  |  |  |  |  |
|  | *Formulate personalized medication, therapy or treatment plans (n = 174)* |  |  |  |  |  |
|  | Extremely Unlikely |  | 3 | 1.7% | **Mean:** | 3.62 |
|  | Unlikely |  | 21 | 12.1% | **Median:** | 4 |
|  | Neutral |  | 39 | 22.4% | **Mode:** | 4 |
|  | Likely |  | 87 | 50.0% | **Interpretation:** | Neutral – Likely |
|  | Extremely Likely |  | 24 | 13.8% |  |  |
|  |  |  |  |  |  |  |
|  | *Evaluate when to refer to specialist (n = 174)* |  |  |  |  |  |
|  | Extremely Unlikely |  | 10 | 5.7% | **Mean:** | 3.23 |
|  | Unlikely |  | 28 | 16.1% | **Median:** | 3 |
|  | Neutral |  | 56 | 32.2% | **Mode:** | 4 |
|  | Likely |  | 72 | 41.4% | **Interpretation:** | Neutral – Likely |
|  | Extremely Likely |  | 8 | 4.6% |  |  |
|  |  |  |  |  |  |  |
|  | *Provide empathetic care (n = 174)* |  |  |  |  |  |
|  | Extremely Unlikely |  | 79 | 45.4% | **Mean:** | 1.78 |
|  | Unlikely |  | 63 | 36.2% | **Median:** | 2 |
|  | Neutral |  | 24 | 13.8% | **Mode:** | 1 |
|  | Likely |  | 8 | 4.6% | **Interpretation:** | Unlikely |
|  | Extremely Likely |  | 0 | 0.0% |  |  |
|  |  |  |  |  |  |  |
